# Endophytic Diversity in Sicilian Olive Trees: Identifying Optimal Conditions for a Functional Microbial Collection

**DOI:** 10.3390/microorganisms13071502

**Published:** 2025-06-27

**Authors:** Dalila Crucitti, Stefano Barone, Salvadora Navarro-Torre, Paola Quatrini, Francesco Carimi, Tiziano Caruso, Davide Pacifico

**Affiliations:** 1 Institute of Biosciences and Bioresources (IBBR), National Research Council (CNR), Via Ugo La Malfa 153, 90146 Palermo, Italy; francesco.carimi@ibbr.cnr.it (F.C.); davide.pacifico@cnr.it (D.P.); 2 Department of Agricultural, Food and Forest Sciences (SAAF), University of Study of Palermo, Viale delle Scienze, 90128 Palermo, Italy; stefano.barone@unipa.it (S.B.); tiziano.caruso@unipa.it (T.C.); 3 Department of Microbiology and Parasitology, Faculty of Pharmacy, University of Seville, C. Profesor García González 2, 41012 Seville, Spain; 4 Department of Earth and Marine Sciences (DiSTeM), University of Study of Palermo, Via Archirafi 22, 90134 Palermo, Italy; paola.quatrini@unipa.it

**Keywords:** *Olea europaea* L. subsp. *europaea* var. *europaea*, *Olea europaea* L. subsp. *europaea* var. *sylvestris*, olive microbiota, cv. ‘Nocellara del Belice’, cv. ‘Nocellara Etnea’, cv. ‘Nocellara Messinese’, plant growth-promoting microorganisms, antifungal activity

## Abstract

This study aims to identify the optimal conditions—host, plant material, seasonality, and agricultural practices—for isolating and developing a collection of culturable endophytic microorganisms to support sustainable *Olea europaea* L. cultivation. Samples were collected from three Sicilian olive cultivars (‘Nocellara del Belice’, ‘Nocellara Etnea’, and ‘Nocellara Messinese’) and six wild olive accessions across different phenological phases and under organic and conventional agronomic management. Endophytes were isolated from leaves and twigs using a culture-dependent approach, and their taxonomic diversity and plant-growth-promoting (PGP) traits were analyzed. A total of 133 endophytic isolates were identified, spanning bacterial (*Proteobacteria*, *Firmicutes*, and *Actinobacteria*) and fungal (*Ascomycota* and *Basidiomycota*) phyla. Wild olive trees contributed more than cultivated varieties to enriching the diversity and composition of culturable endophyte collection as well as twigs instead of leaves. Winter sampling allowed to implement the taxonomic genera of olive endophyte collection. Both farming systems favored an increase in the composition of microbial collection, though organic farming systems supported greater microbial richness. Functional analysis highlighted key PGP traits in a selection of bacterial isolates, including indole-3-acetic acid and siderophore production, nitrogen fixation, and antifungal activity. *Bacillus* spp. dominated enzymatic activities, such as amylase, protease, and lipase production, as well as antifungal activity against the olive fungal pathogen *Neofusicoccum vitifusiforme*. This research highlights the significant diversity and functional potential of Mediterranean olive endophytes. Our findings emphasize the role of native microbial communities as bio-inoculants, promoting plant growth, nutrient uptake, and disease resistance. These insights lay the groundwork for developing targeted olive-microbial consortia for biocontrol and stress tolerance applications.

## 1. Introduction

The plant microbiota—comprising bacteria, fungi, oomycetes, algae, protozoa, nematodes, and viruses—continuously co-evolves with its host, playing a crucial role in plant growth and health [1]. Among these microorganisms, plant growth-promoting endophytes (PGPEs) are particularly valuable, as they colonize internal plant tissues, enhancing biomass accumulation, nutrient acquisition (such as nitrogen, phosphorus, iron, etc.), and the production of beneficial secondary metabolites. PGPEs also contribute to seed germination, root and shoot development, and overall plant resilience by modulating the host immune system, improving tolerance to abiotic stressors and pathogens [2,3].

These beneficial effects are mediated by a range of bioactive compounds and enzymatic activities, including (i) phytohormones such as indole-3-acetic acid (IAA) and gibberellins, which regulate plant development; (ii) siderophores, which enhance iron bioavailability; (iii) phosphate-solubilizing molecules; (iv) trehalose, which improves drought and salt-stress tolerance; (v) hydrolytic enzymes that facilitate bacterial entry into plant tissues; (vi) 1-aminocyclopropane-1-carboxylic acid (ACC) deaminase, which reduces ethylene levels and mitigates abiotic stress; (vii) nitrogen-fixing enzymes; (viii) enzymes capable of degrading complex organic compounds; and (ix) antimicrobial compounds that suppress phytopathogens [4,5]. However, the effectiveness of endophytes depends on microbial strain, plant genotype, physiological state, environmental conditions, and external stressors, making plant-microbiota interactions highly dynamic and complex [6,7,8].

The olive tree (*Olea europaea* L.), a key species in Mediterranean agriculture, is increasingly vulnerable to biotic and abiotic stressors due to shifting climatic conditions and evolving cultivation practices [9]. Italy, the second-largest olive producer in Europe [10], possesses a rich genetic heritage, with 25 officially recognized cultivars in Sicily alone [11]. Despite the economic and ecological importance of olive cultivation, studies on the culturable microbial diversity of the olive phyllosphere remain limited [12,13,14,15]. Most research has focused on how climate, host genotype, plant organ specificity, and seasonal variation influence microbial communities, while investigations into beneficial microorganisms in Italian olive groves have largely concentrated on soil-dwelling microbiota [16]. To bridge these gaps, we investigated the culturable fraction of olive phyllosphere microbiota by maximizing the diversity of both plant hosts and environments, i.e., including wild and cultivated accessions of different varieties collected in Sicilian traditional olive districts.

Considering the loss of microbial diversity associated with domestication [17] and the positive influence of manure amendments on the plant-associated microbial community [18], we hypothesized that (a) wild olives harbor a higher endophytic diversity than cultivars, and (b) organic farming practices enhance microbial richness.

This study aims to identify the optimal conditions for establishing a functional microbial collection from the phyllosphere of Sicilian olive cultivars and wild olive trees. Using a culture-dependent approach, we assessed microbial diversity across different host types, plant organs, phenological stages, and farming systems. Selected bacterial isolates were characterized in vitro for their plant growth-promoting properties, enzymatic activity, and antagonistic potential against major olive pathogens, including *Verticillium dahliae* and *Neofusicoccum* spp. By linking microbial functionality to environmental and host-related factors, this research provides critical insights for the development of an optimized microbial collection, enabling targeted applications in sustainable olive production and biological control strategies.

## 2. Materials and Methods

### 2.1. Orchard Site and Sampling

Plant material was collected from five olive orchards (Table 1 and Supplementary Figure S1) under different pedo-climatic conditions, including mild winters and dry summers in southern Sicily, as well as rainfall and strong thermal fluctuations in suburban areas around Mount Etna in eastern Sicily. Three dual-purpose (table and olive oil use) cultivars (*Olea europaea* L. subsp. *europaea* var. *europaea*), namely ‘Nocellara del Belice’ (NB), ‘Nocellara Etnea’ (NE), and ‘Nocellara Messinese’ (NM), were selected from different sites listed in Table 1. Additionally, wild olive accessions (SYLV) were chosen from two sites within the Pisano Forest (SAC—Special Conservation Area, ITA090022), characterized by a lower humid meso-Mediterranean bioclimate and hosting a rich population of *Olea europaea* L. subsp. *europaea* var. *sylvestris* (Table 1). At each sampling site, leaves and twigs were collected from three randomly selected olive trees during four different periods corresponding to phenological phases: winter rest (WD, December 2021–February 2022), flowering (FL, May 2022), fruit set (FS, June–July 2022), and fruit maturation (MAT, October 2022).

### 2.2. Endophyte Isolation

Samples were collected from four distinct branches distributed across the four cardinal points of the canopy of each olive tree, handled with sterile tools, placed in sterile plastic bags, maintained at 4 °C, and processed within 48 h. Leaves and twig tips (7–8 cm) from each sample were separated and randomly divided into two distinct pools of 10 leaves and 6 twigs. Each pool was weighed, washed with tap water, and surface-disinfected through immersion in 70% (*v*/*v*) ethanol for 2 min, 3% (*w*/*v*) sodium hypochlorite for 3 min, and 70% (*v*/*v*) ethanol for 1 min, followed by three rinses of 1 min each in sterile distilled water (SDW). The disinfected samples were then ground in sterile mortars with 5 mL of SDW. Plant extracts (100 µL) were serially diluted and plated on Nutrient Agar (NA, Condalab) and Potato Dextrose Agar (PDA, Condalab) plates. A negative control was prepared by plating an equal volume of the final wash water onto the same media. Plates were incubated at 26 °C in the dark and observed daily. Total colonies were counted and the number of colony-forming units (CFUs) converted to log_10_ CFUs/g of fresh plant material. Bacterial and fungal colonies were initially grouped according to colony morphological features (shape, color, elevation, surface, margin, aerial mycelium, presence of exudate, and growth rate) and microscopic similarity, and at least two representative colonies of each morphotype for each organ and host sample were selected, purified, and maintained in pure cultures at −80 in glycerol (10–50% *v*/*v*).

### 2.3. Identification of Bacterial and Fungal Endophytes

Molecular identification was performed through sequence analysis of PCR-amplified bacterial 16S rDNA genes and fungal internal transcribed spacer (ITS1, 5.8S, ITS2) rDNA regions. Bacterial isolates were cultivated in Nutrient Broth (NB) medium and incubated at 28 ± 2 °C for 24 h. Bacterial genomic DNA was extracted from cell pellets resuspended in 500 µL of TE buffer, enzymatically lysed with Proteinase K (20 mg/mL), and purified using the phenol/chloroform/isoamyl alcohol (25:24:1) method [19]. The extracted DNA was amplified using universal primers (27f and 1492r) following the original PCR protocol [20].

Pure fungal cultures were obtained on PDA plates incubated for seven days at 28 ± 2 °C. Fungal genomic DNA was extracted from 200 µg of scraped aerial mycelium, disrupted with tungsten carbide beads in a Tissue Lyser II (Qiagen, Hilden, Germany) set at 30 Hz for 2 min, following a Cetyl Trimethyl Ammonium Bromide (CTAB) protocol [21]. DNA amplification was performed using the ITS5/ITS4 primer set [22], following the original PCR protocol with an annealing temperature of 56 °C. The amplified products were purified and sequenced by Eurofins Genomics (Ebersberg, Bayern, Germany).

Bacterial and fungal identification was performed using the highest identity score from BLAST 2.15.0 software of the National Center for Biotechnology Information (NCBI) against the NR database. Sequences were deposited in the GenBank database under accession numbers PP506680-PP506717, PV240332-PV240334, PP513240-PP513283, and PP513285-PP513302 (Supplementary Table S1). A percentage identity > 98% with NCBI sequences was accepted for species-level identification, while identities between 95% and 97% were classified at the genus level. Isolates with identity < 95% were designated as “unknown” [23].

### 2.4. Determination of Plant Growth Promotion Properties and Enzymatic Activities of Bacterial Isolates

The production of IAA was evaluated using a colorimetric technique by incubating 3 mL of a liquid culture in NB with L-tryptophan (100 mg/L) and incubated at 28 °C for 72 h with shaking. Cultures were centrifuged for 5 min at 15,000 × *g* and supernatants transferred to glass tubes. Color changing to pink (positive reaction) was verified following the addition of 4 mL of Salkowski’s reagent [24]. The amount of IAA was calculated by measuring the absorbance at 535 nm in a spectrophotometer (ASYS UVM-340 Microplate Reader, Biochrom, Ltd., Cambridge, UK), accordingly to a standard curve appropriately realized.

Siderophores production was evaluated in Chrome Azurol S (CAS) agar plates [25]. Aliquots (100 μL) of bacterial cultures were inoculated in the wells of the CAS plates and incubated for 7 days at 28 °C in darkness. Bacteria that produced siderophores showed an orange halo.

Phosphate solubilization was determined by the presence of a transparent halo around the bacterial cultures (100 μL) inoculated in wells of NBRIP plates (phosphate growth medium from the National Institute of Botanical Research) after 7 days at 28 °C [26].

Nitrogen fixation was assessed by plating strains in a nitrogen-free minimum medium (NFB; [27]) and incubating for 5 days at 28 °C. Bacterial growth indicated the possible ability of the bacteria to fix atmospheric nitrogen.

The formation of biofilms was determined by checking the adhesion capacity of the bacteria in microplates with 12 wells in NB at 28 °C, incubating for 4 days. After incubation, the biofilm formation was observed in the surface and/or bottom of the wells. Then, each well was stained for 20 min with 0.01% crystal violet [28] to evaluate the presence of a ring of biofilm around the well walls.

The ACC deaminase activity was performed as described by Penrose and Glick (2003) [29]. Briefly, bacterial cultures were incubated in a salts minimal broth added with ACC (5 mM) for 24 h at 28 °C by shaking. The α-ketobutyric acid produced was quantified using a standard curve with known concentrations at 540 nm in a spectrophotometer (ASYS UVM-340 Microplate Reader, Biochrom, Ltd., Cambridge, UK), and ACC deaminase activity was expressed in μmoles of α-ketobutyrate per mg of protein per hour.

Enzymatic activities were determined on plates incubated at 28 °C for 7 days, observing the formation of halo around the bacterial biomass. DNAse activity was determined by streaking the isolates on DNA agar plates revealed with 1 M HCl. Amylase activity was performed on starch agar plates (Scharlab, Barcelona, Spain) and revealed with 10 mL lugol. Protease and lipase activities were assessed in casein agar and Tween 80 media, respectively, as described by Harley and Prescott (2002) [30]. Pectinase and cellulase activities were examined as described by Elbeltagy et al. [31]. For pectinase activity, strains plated on ammonium mineral agar plates were revealed with 2% CTAB, and positive bacteria showed a halo. For cellulase activity, strains were plated on solid minimal medium supplemented with yeast extract (0.2%) and carboxymethylcellulose (1%). Plates were developed by covering the plate with 1 mg/mL Congo Red solution for 15 min and decolorizing with 1 M NaCl for 20 min. Finally, chitinase activity was performed as described by Mesa et al. [32] on agar plates containing minimal medium supplemented with colloidal chitin.

### 2.5. Dual Culture Plate Screening for Antagonistic Activity of Endophytic Members of Consortia

Five top-performing bacterial isolates that exhibited a higher number of plant-growth-promoting (PGP) properties (PGP properties ≥ 3 and enzymatic activities ≥ 2), along with the only isolate presenting chitinase activity, were selected. The six isolates were tested in vitro for their antifungal activity against olive fungal pathogens: two no-defoliating strains of *Verticillium dahliae* (Vd-LT and Vd-CS), one strain of *Neofusicoccum parvum* (Np-P4), and one strain of *Neofusicoccum vitifusiforme* (Nv-P3). Three replicate plates were prepared for each bacterial isolate. For screening, round agar plugs of 5 mm diameter of the filamentous fungi were obtained from the margins (active growth zone) of young mycelia grown on PDA and placed at a distance of 21 mm away from the Petri dish edge (standard 90 mm Petri dishes) with fresh nutrient agar. One hundred microliters of bacterial suspension (~10^6^ cells/mL) were inoculated 45 mm away from the fungal plug on the opposite side of the plate. Negative control plates were inoculated only with the filamentous fungi, while a strain of *Trichoderma harzianum* S3, a fungus commonly used as biocontrol agents [33], was inoculated instead of bacteria as positive control. The plates were incubated for 42 days at 25 °C in the dark. Mycelial growth was observed weekly, and images were captured to record the growth. The inhibition percentage was calculated as [(Rc-Rexp)/Rc] × 100%, where Rc represents the longest distance of fungal mycelium from the inoculated fungal plug, and Rexp is the horizontal distance from the inoculated fungal plug towards the bacterial colony, which shows the inhibitory effect [34,35].

### 2.6. Data Analysis

A non-parametric approach was adopted to analyze the culturable endophyte occurrence in leaves and twigs of different olive varieties and wild accessions during the four phenological phases. To investigate the differences in CFU/g, the Kruskal–Wallis statistic with adjustment for ties [36] was adopted and implemented on Minitab statistical software version 22.1.0 [37].

Richness and evenness were calculated to determine the α-diversity of the culturable fungal and bacterial endophytes identified at genus level in different hosts under several environmental factors. To carry out diversity analyses, the PAST 4.17 software tool [38], version 4.17, was used, estimating the diversity indices and the species rarefaction curves in leaves and twigs for each phenological phase based on the Simpson index (1/D). For diversity analyses, only the identified taxonomic genera were considered, excluding the “unknown”.

β-diversity was examined by means of non-metric multidimensional scaling (NMDS), using the Bray–Curtis coefficient and the Jaccard indexes as similarity measures. Analyses were performed using the PAST 4.17 software, and the quality of the results was visualized in the Shepard plots, accepting stress values ≤ 0.2 as goodness of fit. To assess significant differences among endophyte community groups obtained by NMDS plots, *p*-values (significance assumed for *p* ≤ 0.05) were generated by one-way analysis of similarity (ANOSIM) from Bray–Curtis matrices automatically computed in PAST 4.17 software. To assess which taxa are primarily responsible for a difference between groups of samples, the similarity percentage (SIMPER) analysis was implemented on PAST 4.17 software.

Venn diagrams were made using the opensource component for web environment Jvenn (available online: http://jvenn.toulouse.inra.fr, accessed on 30 April 2025).

To obtain information about the functional diversity of fungal strains, the FUNGuild database was used to estimate the trophic modes and guilds of fungi [39]. This tool allows to predict the following primary fungal lifestyles: pathotroph: receiving nutrients at the expense of the host cells and causing disease; saprotroph: receiving nutrients by breaking down dead host cells; symbiotroph: receiving nutrients by exchanging resources with host cells.

## 3. Results

### 3.1. Culturable Endophyte Occurrence in Sicilian Olive Cultivars and Wild Accessions

**Figure 1:**
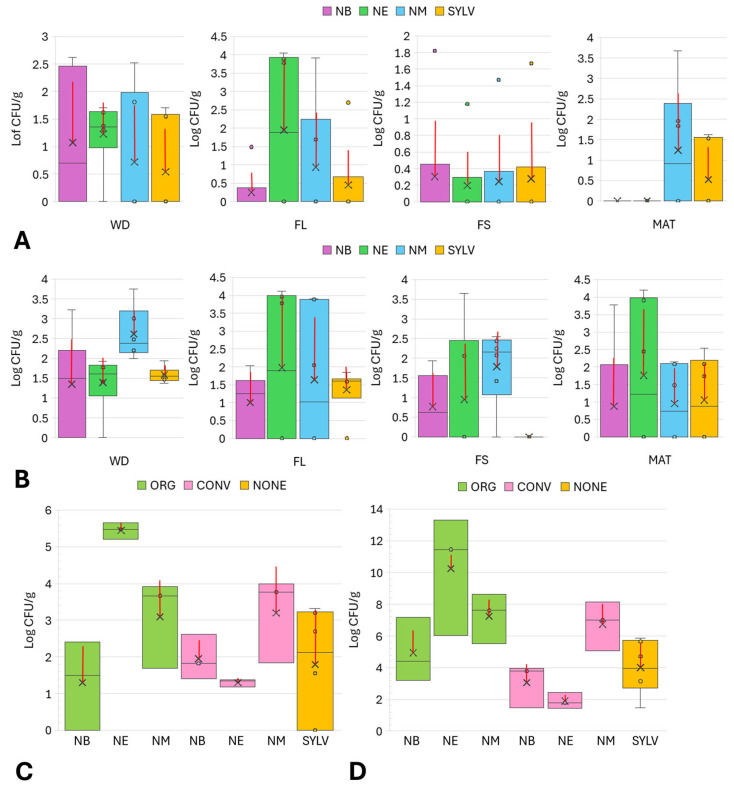
Box plots showing total microbial counts (log_10_ CFU/g) of leaf (**A**,**C**) and twig (**B**,**D**) endophytes isolated from the different cvs. ‘Nocellara del Belice’ (NB), ‘Nocellara Etnea’ (NE), and ‘Nocellara Messinese’ (NM) and Sicilian wild olive trees (SYLV) during the four phenological phases (WD, winter rest; FL, flowering; FS, fruit set; MAT, fruit maturation) (**A**,**B**) and farmed under organic (ORG) and conventional (CONV) management and no agricultural practices (NONE) (**C**,**D**). The central horizontal bars are the medians. The central symbol X corresponds to average values, and the red vertical lines are the standard deviations. Points above and below the whiskers’ upper and lower bounds are outliers.

The presence of bacterial and fungal colonies on NA and PDA media increased during the incubation period, and most of them appeared within 10 days. No colonies were obtained from any dilutions. The absence of colonies was observed on some NA and PDA plates regardless of olive samples, organs and phenological phases, probably due to the low concentration and erratic distribution typical of plant endophytes. Considering the plant material, most of colonies were recovered from twigs compared to occasional microbial isolation from leaves. Twigs enabled the isolation of endophytes from more olive trees along all phenological phases (Figure 1A,B) as well as farming systems (Figure 1C,D).

Within cultivars, the occurrence of culturable endophytes was significantly influenced by twig samples in ‘Nocellara del Belice’ (*p* = 0.036), ‘Nocellara Messinese’ (*p* = 0.007), and wild olive accessions (*p* = 0.038) compared to leaves. The highest value of total microbial count in twigs was observed during fruit maturation in ‘Nocellara del Belice’, ‘Nocellara Etnea’, as well as wild olive trees and during flowering in ‘Nocellara Messinese’ (Figure 1B). Regarding farming systems, samples from olive trees under organic management showed a higher microbial occurrence (Figure 1C,D), which varied significantly in samples from ‘Nocellara Etnea’ (*p* = 0.001) compared to those under conventional management. Among phenological phases, no significant differences were observed in olive cultivar and wild olive tree total microbial count.

### 3.2. Diversity of Culturable Endophytes

From olive leaves and twigs collected during all phenological phases, a total of 133 phenotypically different endophytic bacterial and fungal isolates were selected and identified by 16S rRNA gene and ITS sequencing analysis, respectively. Sequence lengths varied from 474 to 1425 bp for 16S rRNA region and from 417 to 651 bp for the ITS region (Supplementary Table S1).

**Figure 2:**
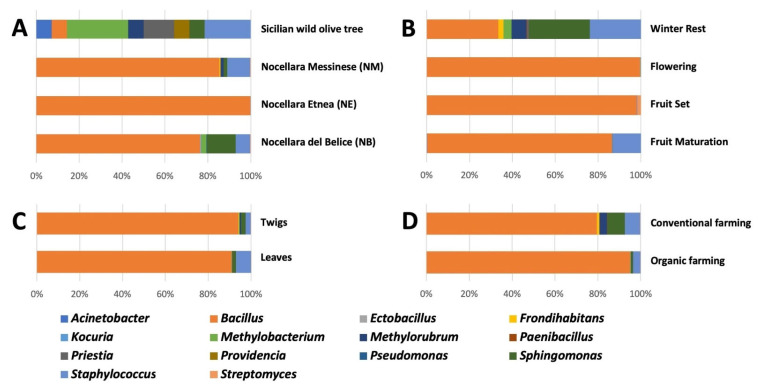
Relative abundance of endophytic bacterial genera in hosts (**A**), phenological stages (**B**), plant organs (**C**), and farming systems (**D**) of olive trees cvs. ‘Nocellara del Belice’ (NB), ‘Nocellara Etnea’ (NE), and ‘Nocellara Messinese’ (NM) and Sicilian wild olive trees (SYLV).

**Figure 3:**
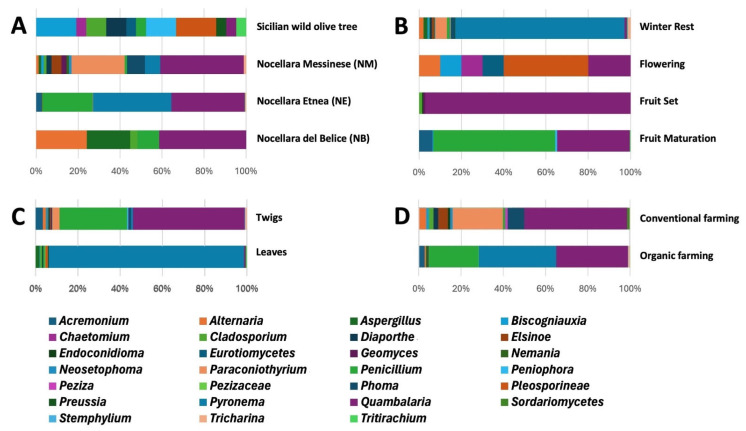
Relative abundance of endophytic fungal genera in hosts (**A**), phenological stages (**B**), plant organs (**C**), and farming systems (**D**) of olive trees cvs. ‘Nocellara del Belice’ (NB), ‘Nocellara Etnea’ (NE), and ‘Nocellara Messinese’ (NM) and Sicilian wild olive trees (SYLV).

The 60 bacterial isolates identified were affiliated to three phyla (Actinobacteria, Firmicutes, and Proteobacteria) and mainly associated most with the genera *Bacillus* (43.3%), *Staphylococcus* (18.3%), and *Sphingomonas* (10.0%) (Figure 2). The 68 fungal isolates identified were assigned to two phyla (Ascomycota and Basidiomycota) and affiliated to the genera *Quambalaria* (17.4%), *Alternaria* (10.1%), and *Biscogniauxia* (8.7%) (Figure 3). The five isolates that showed a sequence identity ≤ 94.7% were excluded from analyses (Supplementary Table S1). The microbial diversity (high or low) found in each sample was independent of the overall number of microorganisms present, regardless of host, organ, phenological phase, or farming system.

Considering olive hosts, Sicilian wild olive trees contributed most to the diversity of bacterial and fungal endophyte collection (Figure 2A and Figure 3A) and significantly to microbial composition (R = 0.562; *p* = 0.008) compared to cultivated varieties that showed a more homogeneous endophytic composition (few different dominant genera with individuals uniformly distributed), with minor differences (Table 2; Supplementary Figure S2). Rarefaction curves showed that the probability to enrich the microbial collection of endophytes can be further increased by analyzing a larger number of wild olive samples, while for cultivated olive trees, the plateau seems to be reached (Supplementary Figure S3).

**Figure 4:**
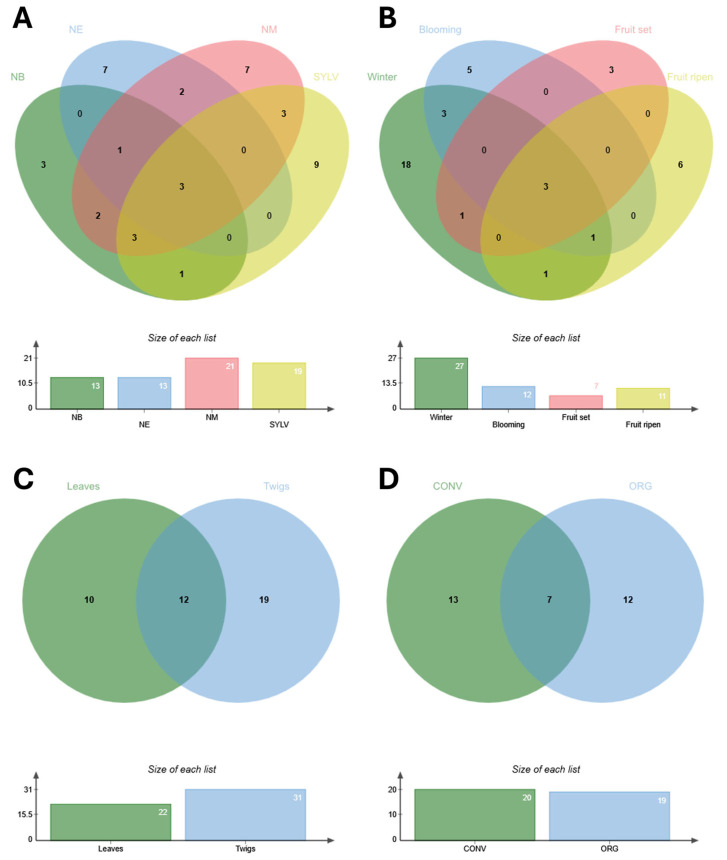
Venn diagram of shared and specific microbial genera from different cvs. ‘Nocellara del Belice’ (NB), ‘Nocellara Etnea’ (NE), and ‘Nocellara Messinese’ (NM) and Sicilian wild olive trees (SYLV); (**A**) phenological phases (**B**), plant organs (**C**), and farming systems (CONV, conventional; ORG, organic) (**D**).

Nine microbial genera (*Acinetobacter*, *Chaetomium*, *Eurotiomycetes*, *Peniophora*, *Pleosporineae*, *Preussia*, *Priestia*, *Providencia*, and *Tritirachium*) derived exclusively from wild plants (Figure 4A). Endophytes belonging to ten genera, i.e., *Bacillus, Methylobacterium*, *Methylorubrum*, *Sphingomonas*, *Staphylococcus*, *Biscogniauxia*, *Cladosporium*, *Diaporthe*, *Penicillium,* and *Quambalaria*, were isolated both from wild olive accessions and at least one of the three cultivars of ‘Nocellara’, representing ~25% of the endophytic communities (Supplementary Table S1). At species level, the bacterial endophytes *Ectobacillus funiculus*, *Pseudomonas savastanoi*, and *Streptomyces bryophytorum* and the fungal ones, namely *Aspergillus stella-maris* and *Cladosporium sphaerospermum*, were isolated only from leaf samples of the cultivar ‘Nocellara del Belice’, which clustered separately from the samples of other cultivars (Supplementary Figure S2A,B). Although differences in pairwise comparisons of microbial communities from different hosts were not significant (*p* > 0.05), the SIMPER analysis showed that the species of the genus *Bacillus* and secondarily those of the genus *Pyronema* and the species *Sphingomonas paucimobilis* contributed greatly to determining the differences among hosts, influencing more the diversification of isolation of culturable olive endophytes. Despite this diversity, a putative microbial core, defined as the community systematically associated with a given host plant [40], of culturable endosphere-associated microorganisms from Sicilian olive trees can be attributed to the presence of the genera *Bacillus*, *Staphylococcus,* and *Quambalaria*.

In almost all phenological phases, microbial isolates from wild olive trees enriched culture collection diversity and composition more than those from cultivated varieties (Figure 2B and Figure 3B, Supplementary Table S2). Among all samples, the highest number of microbial genera was isolated during the winter rest (Figure 4B), with a prevalence of fungal endophytes. The endophytic genera *Bacillus*, *Quambalaria,* and *Staphylococcus* were isolated during all sampling periods.

A wider diversity of endophytic isolates was reached in twigs rather than leaves both along all phenological phases and different olive hosts (Table 2 and Supplementary Table S2). Twig samples were richer on genera (Figure 2C, Figure 3C, and Figure 4C); about 35.7% of bacterial (*Acinetobacter*, *Frondihabitans*, *Kocuria*, *Priestia,* and *Providencia*) and 55.5% of fungal (*Alternaria*, *Acremonium*, *Chaetomium*, *Diaporthe*, *Dydimella*, *Elsinoe*, *Geomyces*, *Nemania*, *Neosetophoma*, *Paraconiothyrium*, *Peniophora*, *Peziza*, *Phoma*, *Stemphylium,* and *Tricharina*) genera were isolated only from that plant material, mainly from cv. ‘Nocellara Messinese’. More than 20% of both bacterial and fungal genera were isolated from only leaves (*Ectobacillus*, *Pseudomonas*, *Paenibacillus*, *Endoconidioma*, *Lybertasomyces*, *Preussia*, *Pleosporinae*, *Sordariomycetes*, *Pezizaceae,* and *Tritirachium*). Almost 30% of microbial genera (*Bacillus*, *Methylobacterium*, *Methylorubrum*, *Sphingomonas*, *Staphylococcus*, *Streptomyces*, *Aspergillus*, *Biscogniauxia*, *Cladosporium*, *Penicillium,* and *Quambalaria*) were recovered from both plant organs.

The microbial diversity of the culturable endophytic collection was considerably increased by the contribution of microbial communities from samples of the organically farmed cv. ‘Nocellara del Belice’ as well as cultivars ‘Nocellara Etnea’ and ‘Nocellara Messinese’, farmed under conventional practices (Supplementary Table S3). Regarding taxonomical genera, the endophyte collection was enriched both from samples collected under organic (12) and conventional (13) practices (Figure 4D). The bacterial genera (*Ectobacillus*, *Kocuria*, *Methylobacterium,* and *Pseudomonas*) and fungal genera (*Aspergillus*, *Geomyces*, *Nemania*, *Pezizaceae* sp., *Pyronema*, *Stemphylium,* and *Tricharina*) associated with organic farming systems were different from the genera (bacterial: *Frondihabitans*, *Methylorubrum,* and *Paenibacillus*; fungal: *Biscogniauxia*, *Cladosporium*, *Diaporthe*, *Elsinoe*, *Endoconidioma*, *Neosetophoma*, *Paraconiothyrium*, *Peziza*, and *Sordariomycetes* sp.) associated with conventional managements (Figure 2D and Figure 3D). Only seven microbial genera (*Alternaria*, *Bacillus*, *Penicillium*, *Quambalaria*, *Sphingomonas*, *Staphylococcus,* and *Streptomyces*) were found to be widespread in cultivars farmed under both agricultural practices. Endophyte isolates from samples of each cultivar under organic management were taxonomically different from those isolated from conventional management, but no clear differentiation and no significant dissimilarities of two groups (ORG and CONV) was observed by NMDS ordination (Supplementary Figure S2C,D) and ANOSIM analysis. *Bacillus*, *Sphingomonas*, *Alternaria,* and *Quambalaria* from cv. ‘Nocellara del Belice’; *Staphylococcus* and *Quambalaria* from cv. ‘Nocellara Etnea’; and *Bacillus* and *Staphylococcus* from cv. ‘Nocellara Messinese’ were the microbial genera found in common between the two farming systems.

### 3.3. Characterization of Endophytic Bacteria from Olive Leaves and Twigs

#### 3.3.1. Plant Growth-Promotion Properties and Enzymatic Activities

A total of 45 bacterial isolates were characterized to evaluate the presence of plant growth-promotion traits, and about 90% of them, representing those with greater growth capacity in vitro, showed PGP properties (Supplementary Table S4) and enzymatic activities (Supplementary Table S5). All isolates showed at least one of the screened characteristics, and 65% of them exhibited from five to ten PGP traits concurrently. The most common PGP properties were siderophore production (80%), biofilm formation (77.5%), and nitrogen fixation (65%). Bsp_GIAL05R strain (*Bacillus* sp.) showed the highest production of siderophores according to the diameter of the halo in CAS medium (Supplementary Table S4), and Bm_GIAL02R (*Bacillus megaterium*), Msp_SYLV02F (*Methylobacterium* sp.), and Pv_SYLV05R (*Providencia vermicola*) were the best phosphate solubilizers (Supplementary Table S4). A high number of isolates (26) were able to grow in N-free medium. The strain Pv_SYLV05R (*Providencia vermicola*) produced the highest concentration of IAA, more than 9 mg/L (Supplementary Table S4). Bacterial isolates did not show biological ACC deaminase activity in vitro. Among the seven enzymatic activities studied, amylase (57.5%), lipase (52.5%), and DNase (52.5%) were the most represented (Supplementary Table S5). Protease synthesis was found in 15 endophytes, and four strains of *Bacillus* genus (Bsp_NMC03R, Bsp_NMC03F, Bm_GIAL02Rb, and Bsp_NEB03RIII) showed a strong activity (Supplementary Table S5). Cellulase, pectinase, and chitinase activities were detected in a low number of isolates (three to seven).

#### 3.3.2. Antagonistic Activities

**Figure 5:**
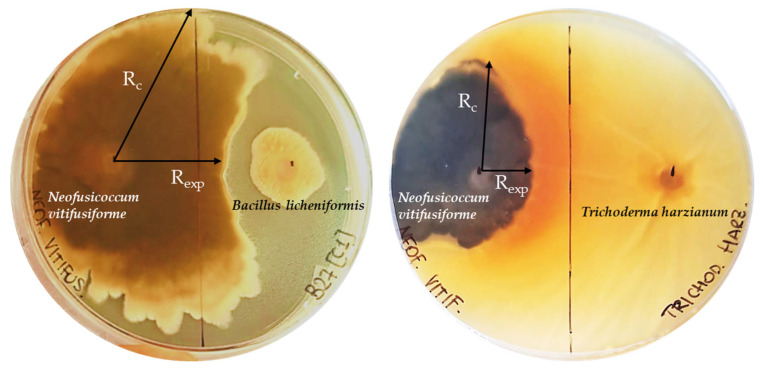
Representation of the dual culture assay showing antifungal activity of *Bacillus licheniformis* Bl_SYLV02R and *Trichoderma harzianum* S3 against the fungal pathogen *Neofusicoccum vitifusiforme* (Nv-P3) and how the inhibition was measured. R_c_ represents the longest distance of fungal mycelium growth from the inoculated fungal plug, and R_exp_ is the horizontal distance from the inoculated fungal plug towards the screened isolates.

Among the screened bacterial isolates, *Bacillus licheniformis* Bl_SYLV02R displayed inhibitory activity against the fungal pathogen *Neofusicoccum vitifusiforme* (Nv-P3) in dual-plate assay (Figure 5). A mycelial inhibition percentage equal to 40.3% was recorded compared to that of the positive control, explained by the 60% inhibition by *Trichoderma harzianum* S3 against the same pathogenic fungus (Supplementary Table S6). It is noteworthy that the other different tested species within the *Bacillus* genus (*Bacillus* sp. and *Bacillus marisflavi*) did not exhibit the same inhibition capability. No inhibitory activity was observed against the two no-defoliating strains of *Verticillium dahliae* (Vd-LT and Vd-CS) and the fungus *Neofusicoccum parvum* (Np-P4) by *Bacillus licheniformis* Bl_SYLV02R or by the other bacterial isolates against all the pathogenic fungi considered.

### 3.4. Trophic Mode of Endophytic Fungi

Fungal taxonomic functional analyses by FUNGuild classified the fungal strains into different trophic modes (Supplementary Table S7). The most common trophic mode was pathotroph–saprotroph–symbiotroph (41.2% of identified isolates), followed by pathotroph (13.2%) and saprotroph (11.7%) (Supplementary Table S7). The main trophic mode was mainly represented by endophytes, animal and plant pathogens, and wood saprotrophs (Supplementary Table S7). The pathotroph group was exclusively represented by the plant pathogen *Quambalaria cyanescens,* most frequently isolated under conventional farming. The species *Endoconidioma populi*, *Geomyces* sp., *Libertasomyces platani*, *Nemania serpens*, *Paraconiothyrium brasiliense,* and *Preussia minima* were classified as wood and plant saprotroph. The endophytes *Neosetophoma* sp., *Pyronema* sp., and *Tricharina* sp. and the dung saprotrophs *Peziza* sp. and *Pezizaceae* sp. belonged to the saprotroph–symbiotroph group and were isolated only during the winter rest and mostly under organic farming systems.

## 4. Discussion

The beneficial interaction between plants and their phyllosphere endophytes is well recognized, yet relatively few studies have explored the functional potential of endophytic communities associated with olive trees in Mediterranean environments. This study aimed to establish a collection of beneficial microorganisms for potential application in olive cultivation to enhance plant tolerance to abiotic and biotic stresses. Focusing on the culturable fraction of the olive endophytic microbiota, we leveraged the plant’s natural selection of symbiotic partners to isolate microorganisms with multiple PGP traits, ultimately identifying candidates for future bioinoculant development. To maximize microbial diversity, we sampled different olive cultivars representative of the local germplasm, cultivated under varying pedoclimatic conditions and either organic and conventional farming systems, and including wild olive accessions, across different phenological stages. A sampling protocol including the minimal standard number of biological replicates (three olive trees) was adopted, as applied in similar studies [41]. To recover the culturable fraction of microbial communities, we prepared leaf and twig pools prior to surface disinfection. This procedure is a standard method for isolating endophytes from woody crops [42], ensuring efficient sample processing within 24 h. It is important to note that this work does not seek to provide a comprehensive overview of the entire olive endophytic microbiota but rather to define optimal conditions for exploring the culturable fraction associated with some of the most widespread Sicilian varieties, identifying potential key taxa under different agroecological conditions.

For microbial isolation and maintenance, two general-purpose media (NA and PDA) were selected as widely used to isolate endophytes from plants [13,43,44,45,46,47,48]. As highlighted by Eevers et al. [49], complex media containing glucose and yeast extract foster higher endophyte cultivability than minimal media. Despite this, colony counts remained low across both media, irrespective of sample origin. Isolation efficiency likely depends not only on culture conditions and sterilization protocols but also on the intrinsic rarity, uneven distribution, and varied growth capacities of plant endophytes under aseptic conditions [50]. Moreover, fewer colonies are generally expected when surface disinfection is applied on plant tissues prior to strain isolation compared to soil samples. The use of additional selective, enriched, as well as oligotrophic media could improve future recovery rates as well as allowing the isolation of slow-growing taxa.

Considering that a species-level identification needs to combine morphological and muti-loci phylogenetic analyses [51], particularly for bacterial and fungal isolates, identification was limited at genus level for diversity analyses. The single target molecular identification used in this work did not allow us to clarify the taxonomic position of “unknown” isolates, which could represent novel taxa beneficial to the olive host. Future phylogenetic studies could better define their taxonomic position. Lacking sufficient genetic information and experimental evidence for pathotroph isolates, it was not possible to evaluate their real pathogenicity.

Many isolates recovered in this study have been previously reported as olive endophytes with plant-beneficial properties [14,46,52,53,54,55]. Notably, genera such as *Ectobacillus*, *Geomyces*, and *Tritirachium* were identified in olive trees for the first time, though they have been described as endophytes in other plant species [52,56]. According to the literature and based on our results, the isolates Ef_GIAL02F, Geosp_NMB03R, and Tritb_SYLV06F could play a role in thermal regulation, adaptation, and plant protection through enzyme and antibiotic production. The microbiota of Sicilian olive cultivars was predominantly composed of *Proteobacteria*, *Firmicutes*, and *Actinobacteria*, alongside fungal members of the *Ascomycota* phylum. Similar profiles have been reported in previous studies on Mediterranean olive trees [14,57,58], with *Proteobacteria*—particularly Pseudomonadales—frequently dominating, along with *Actinobacteria*, *Firmicutes*, *Bacteroidetes*, and other *Proteobacteria* isolated from cultivated and wild olive xylem sap [57]. Similarly, a higher prevalence of *Proteobacteria* was observed in Spanish cultivars and Portuguese wild olive trees [58]. The recovered fungal community was rich and largely composed of Dothideomycetes and Sordariomycetes, in agreement with earlier findings on Portuguese and Spanish olive orchards [13,59].

By accounting for host type (cultivated vs. wild), plant organs, phenological stages, and farming systems, this study sought to identify optimal conditions to build a representative collection reflecting the natural diversity of olive endophytes. Previous research by Müller et al. [58] compared the microbiota of cultivated and wild olive trees across Mediterranean regions, revealing that both genotype and geographical origin shape bacterial endophyte communities. Their analysis showed that wild olives clustered with cultivars from the same region, yet distinct taxonomic groups differentiated cultivated from wild trees. Our findings align with this pattern: Sicilian wild olive accessions formed distinct groupings compared to domesticated varieties while maintaining a closely related microbiota characterized by the presence of the class Eurotiomycetes, the family Pleosporineae, and the genera *Acinetobacter*, *Chaetomium*, *Peniophora*, *Preussia*, *Priestia*, *Providencia*, and *Tritirachium*. This result supports the hypothesis that domestication has progressively reduced microbial biodiversity. Historically, the selection of cultivated plants has prioritized agronomic and productive traits, often overlooking their capacity to establish beneficial relationships with soil and plant-associated microorganisms. Modern varieties, bred for high yields under intensive management, may have lost traits involved in hosting growth-promoting and pathogen-antagonistic endophytes. In contrast, wild relatives typically maintain richer and more diverse microbial communities [17]. Despite this, a conserved “core” microbiota persists across both wild and cultivated olives, which is functionally essential for plant fitness through genes acquired by evolutionary selection [60]. Within this core, the keystone taxa, i.e., highly connected taxa that influence community structure and function irrespective of their abundance across space and time [61], play a crucial role in ecosystem stability, nutrient cycling, pathogen suppression, and plant health [40]. Meanwhile, less abundant and transient satellite taxa contribute to disease resistance, resilience, and functional redundancy [62]. In our study, while ‘Nocellara’ and wild olive trees shared a core microbiota represented by *Bacillus*, *Staphylococcus,* and *Quambalaria*, wild accessions harbored greater microbial diversity, likely reflecting their adaptation to more heterogeneous and unmanaged environments.

Regarding plant organs, twigs hosted a higher number of taxa than leaves, a trend well documented across plant species. A meta-analysis by Harrison and Griffin [63] confirmed the predominance of endophytic fungi and bacteria in woody stems over other tissues. This was also evident in our study, with most culturable fungal species isolated from twigs, likely due to the richer presence of structural carbohydrates that promote fungal colonization [64]. Seasonal variation in olive endophytic communities remains poorly explored [12,46,65]. In line with Abdelfattah et al. [65], we recorded peak species richness and diversity during the winter rest period, dominated by fungal isolates—a pattern also reported for other Mediterranean woody crops [44]. According to results reported by Gomes et al. [13], climatic conditions typical of Sicilian winters, including higher winds, rainfall, and marked temperature–humidity fluctuations, likely enhance microbial dispersal and colonization. Notably, microbial composition shifted during sampling, favoring *Bacillus* and pigmented taxa, groups widely acknowledged for their stress tolerance and ecological versatility (mesophilic nature and endospore formation), making them valuable biofertilizers [66,67,68]. Most studies on farming practices and plant microbiota have focused on soil communities, but relatively few have addressed endophytes under different cultivation systems. Rhizosphere diversity is known to depend on soil properties, plant species, and management system, often increasing under organic practices [69]. Supporting this, Sofo et al. [70] and Wentzien et al. [18] found higher microbial abundance and diversity in organic olive orchards. Although focused on phyllosphere endophytes, our work also revealed greater microbial richness and abundance among plants under organic management.

While studies have begun to describe the olive root microbiome and its role in enhancing plant physiology [53,71,72,73], knowledge of the functional traits of olive phyllosphere endophytes remains limited. In this study, bacterial endophytes exhibited multiple PGP traits, including siderophore production, biofilm formation, auxin (IAA) synthesis, nitrogen fixation, and hydrolytic enzyme activity. Iron is a key nutrient for plant metabolism [74,75]. Microorganisms assist in its acquisition by producing siderophores that chelate ferric iron [76]. In our collection, 76.2% of bacterial isolates produced siderophores, with *Bacillus*, *Sphingomonas*, and *Staphylococcus*, mainly isolated from organic management, being predominant. While siderophore production by *Bacillus* and *Staphylococcus* has been documented in the olive rhizosphere [73], this is the first report of siderophore-producing *Sphingomonas* endophytes in olives, expanding their documented plant-beneficial roles [77]. Over 80% of isolates formed biofilms, with *Sphingomonas*, *Bacillus*, *Providencia*, and *Kocuria* showing the strongest capacity, in agreement with their known contribution to stress resilience and crop productivity [78,79]. Auxin IAA production was observed in 38% of isolates mostly collected in organic olive orchards and wild accessions, with *Priestia*, *Bacillus*, and *Providencia* being the most active producers. These findings are consistent with reports associating these genera with plant growth promotion, nitrogen fixation, and biocontrol activities against phytopathogenic nematodes and fungi [80,81,82,83,84,85]. Nitrogen fixation capacity was demonstrated by over 60% of isolates, including *Acinetobacter*, *Bacillus*, *Paenibacillus*, *Priestia*, *Pseudomonas*, *Sphingomonas*, and *Staphylococcus*, which are generally found regardless of management practices. These genera have previously been recognized as nitrogen fixers and potential biofertilizers [77,86,87,88,89]. Hydrolytic enzymes such as DNase, amylase, cellulase, chitinase, lipase, protease, and pectinase, involved in microbial colonization and plant protection, were widely produced among isolates. *Bacillus* showed the greatest enzymatic activity, followed by *Frondihabitans*, *Paenibacillus*, *Pseudomonas*, and *Sphingomonas*. *Bacillus* and *Pseudomonas* are the most prevalent PGP bacteria for enzymatic activities, with *Bacillus*-based biostimulants being widely commercialized due to their efficient metabolite synthesis [90]. The ecological role of *Bacillus* endophytes can include mechanisms of competitive exclusion such as resource competition, competition for attachment sites, and the production of antimicrobial compounds. The dominant presence of the genus *Bacillus* could be attributed to its ecological trade-offs as well as to selective recruitment by the host. *Bacillus* protein toxins and metabolites enhance colonization while exerting antagonistic effects on plant pathogens and insect pests [91,92]. Antifungal, antibacterial, and algicidal activities of siderophores are well documented [93,94,95,96,97]. *Bacillus licheniformis* lipopeptides, cell lytic enzymes, and siderophores contribute to the inhibition of pathogenic fungi [98,99]. In this study, *Bacillus* Bl_SYLV02R, characterized by siderophore and biofilm production as well as multiple enzymatic activities, showed significant antifungal activity against the olive pathogen *Neofusicoccum vitifusiforme*. Further studies are warranted to elucidate the mechanisms underlying this antifungal capacity and to assess the performance of these PGP isolates in planta, both individually and as microbial consortia.

## 5. Conclusions

In conclusion, wild olive trees, twig samples, and winter sampling were the optimal conditions to build a variegated collection of Sicilian olive culturable endophytes. Twigs hosted significantly higher endophyte abundance than leaves, and uncultivated olive trees could be considered as custodians of higher biodiversity. Organic management of olive yards seems promising for a richer functional endophyte collection, although further studies should be performed. Potential plant growth-promoting features for nutrient uptake, plant growth promotion, and protection against fungal diseases were demonstrated by bacterial endophytes, which would improve the overall fitness of the host plants.

The potential role of these Sicilian olive endophyte communities needs to be further investigated, evaluating their possible use as native microbial consortia in sustainable cultivation practices. Future applications for sustainable agriculture could be enhanced by integrating the native microbial communities of both cultivated and wild olives, selecting microbial consortia for plant growth promotion, biocontrol, and tolerance/resistance to biotic stresses.

## Figures and Tables

**Table 1:** List of olive trees, cultivars, and wild accessions with the corresponding sampling sites and farming systems; NB: ‘Nocellara del Belice’; NE: ‘Nocellara Etnea’; NM: ‘Nocellara Messinese’; SYLV: wild olive trees. A map of sampling sites is also available in Supplementary Figure S1.

Olive Tree ID	Olive Host	Olive Yards	GPS Coordinates	Farming System
GIAL01 GIAL02 GIAL03	NB	Castelvetrano (Trapani, South-West Sicily)	N 37°41′56.1″; E 12°47′33.1″	Organic
GIAL04 GIAL05 GIAL06	NB	Castelvetrano (Trapani, South-West Sicily)	N 37°41′49.2″; E 12°48′21.9″	Conventional
NEB01 NEB02 NEB03	NE	Motta Sant’Anastasia (Catania, North-East Sicily)	N 37°31′15.8″; E 14°56′21.1″	Organic
NEC04 NEC05 NEC06	NE	Adrano (Catania, North-East Sicily)	N 37°41′21.6″; E 14°50′25.1″	Conventional
NMB01 NMB02 NMB03	NM	Motta Sant’Anastasia (Catania, North-East Sicily)	N 37°31′15.8″; E 14°56′21.1″	Organic
NMC01 NMC02 NMC03	NM	Modica (South-East Sicily)	N 36°50′04.4″; E 14°46′57.4″	Conventional
SYLV01 SYLV02 SYLV03	SYLV	Pisano forest (Buccheri, East Sicily)	N 37°10′58.9″; E 14°52′28.4″	None
SYLV04 SYLV05 SYLV06	SYLV	Pisano forest (Buccheri, East Sicily)	N 37°10′76.2″; E 14°52′28.2″	None

**Table 2:** Diversity indices of endophytes associated with leaves (L) and twigs (T) of different olive hosts (NB, ‘Nocellara del Belice’; NE, ‘Nocellara Etnea’; NM, ‘Nocellara Messinese’; SYLV, wild olive accessions).

Diversity Indexes	NB-L	NB-T	NE-L	NE-T	NM-L	NM-T	SYLV-L	SYLV-T
Taxa (genera)	7	10	6	10	5	21	9	15
Individuals	33	398	1204	2002	397	853	16	19
Dominance	0.428	0.592	0.621	0.590	0.633	0.662	0.108	0.029
Simpson (1-D)	0.572	0.408	0.380	0.410	0.367	0.338	0.892	0.971
Shannon	1.282	0.963	0.589	0.796	0.605	0.935	2.243	2.993
Equitability	0.659	0.418	0.329	0.346	0.376	0.307	1.021	1.105
Chao-1	9.91	10.50	12	20	6.99	36.98	16.03	35.84

## Data Availability

The original data presented in the study are openly available in the GenBank NCBI at https://www.ncbi.nlm.nih.gov/nuccore/PP506680.1/, accessed on 22 June 2025.

## Supplementary Materials

The following supporting information can be downloaded at https://www.mdpi.com/article/10.3390/microorganisms13071502/s1. Figure S1: Sampling sites; Figure S2: NMDS plots; Figure S3: Rarefaction curves; Table S1: Endophyte molecular identification; Table S2: Diversity indices during phenological phases; Table S3: Diversity indices in farming systems; Table S4: PGP properties; Table S5: Enzymatic activities; Table S6: Antagonistic abilities; Table S7: Functional diversity of fungal endophytes isolated from Sicilian olive tree, according to FUNGuild database.
