# Supplementary material for: Endophytic Diversity in Sicilian Olive Trees: Identifying Optimal Conditions for a Functional Microbial Collection
Source: Microorganisms. 2025 Jun 27;13(7):1502. doi: 10.3390/microorganisms13071502 (PMC12298726; doi:10.3390/microorganisms13071502)
Supplement: Supplementary file 1 [file microorganisms-13-01502-s001.zip › Supplementary Figure S2_NMDS plots.pdf]

Supplementary Figure S2 Non-metric multidimensional scale (NMDS) plots corresponding to the clustering of endophyte communities from different olive hosts grouped by A) and B) plant organs (L, leaves; T, twigs), C) and D) farming system. Cluster analysis was performed with two different community similarity measures, namely, Bray–Curtis coefficient (panels on the left) and Jaccard's index (panels on the right). Kruskal's stress values less than 0.2 represent good ordination plots.

A	B
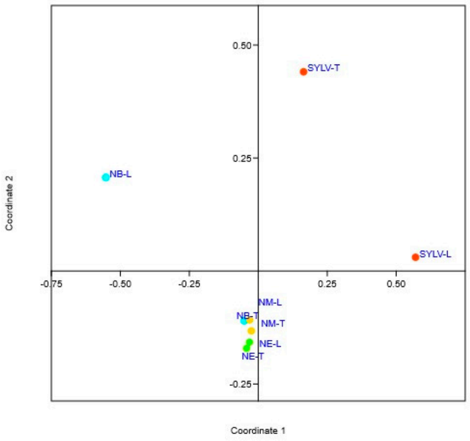	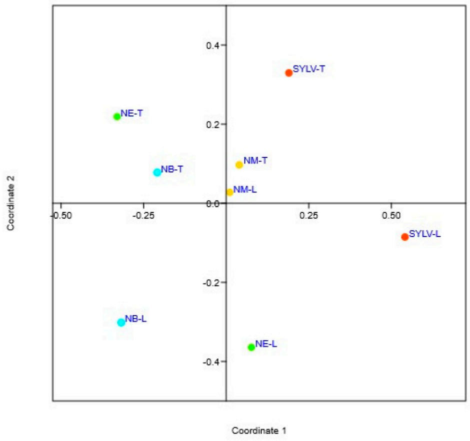
Stress: 0.11	Stress: 0.17
**C**	**D**
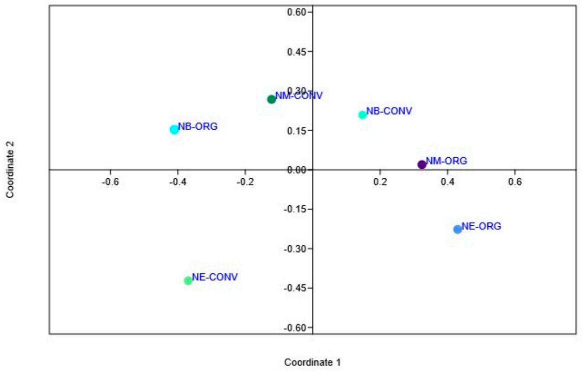	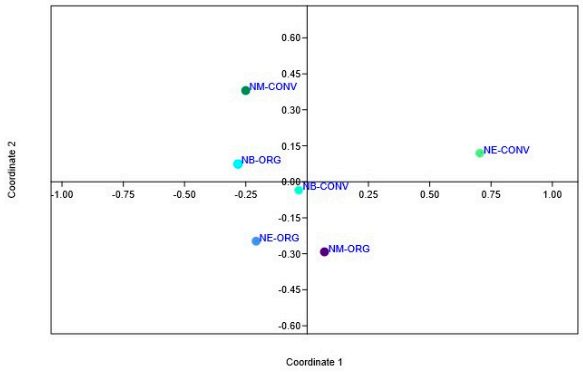
Stress: 0.00	Stress: 0.08
