# Supplementary material for: Endophytic Diversity in Sicilian Olive Trees: Identifying Optimal Conditions for a Functional Microbial Collection
Source: Microorganisms. 2025 Jun 27;13(7):1502. doi: 10.3390/microorganisms13071502 (PMC12298726; doi:10.3390/microorganisms13071502)

Supplementary Figure S3 Species rarefaction curves of endophytes isolated from each plant tissue (leaves, blue; twigs, green) collected from cv. Nocellara del Belice (NB), cv. Nocellara etnea (NE), cv. Nocellara messinese (NB) and wild olive accessions (SYLV). Curves were obtained using the individual rarefaction analysis in PAST 4.17 software, setting Simpson 1/D (Hill's number) as index.

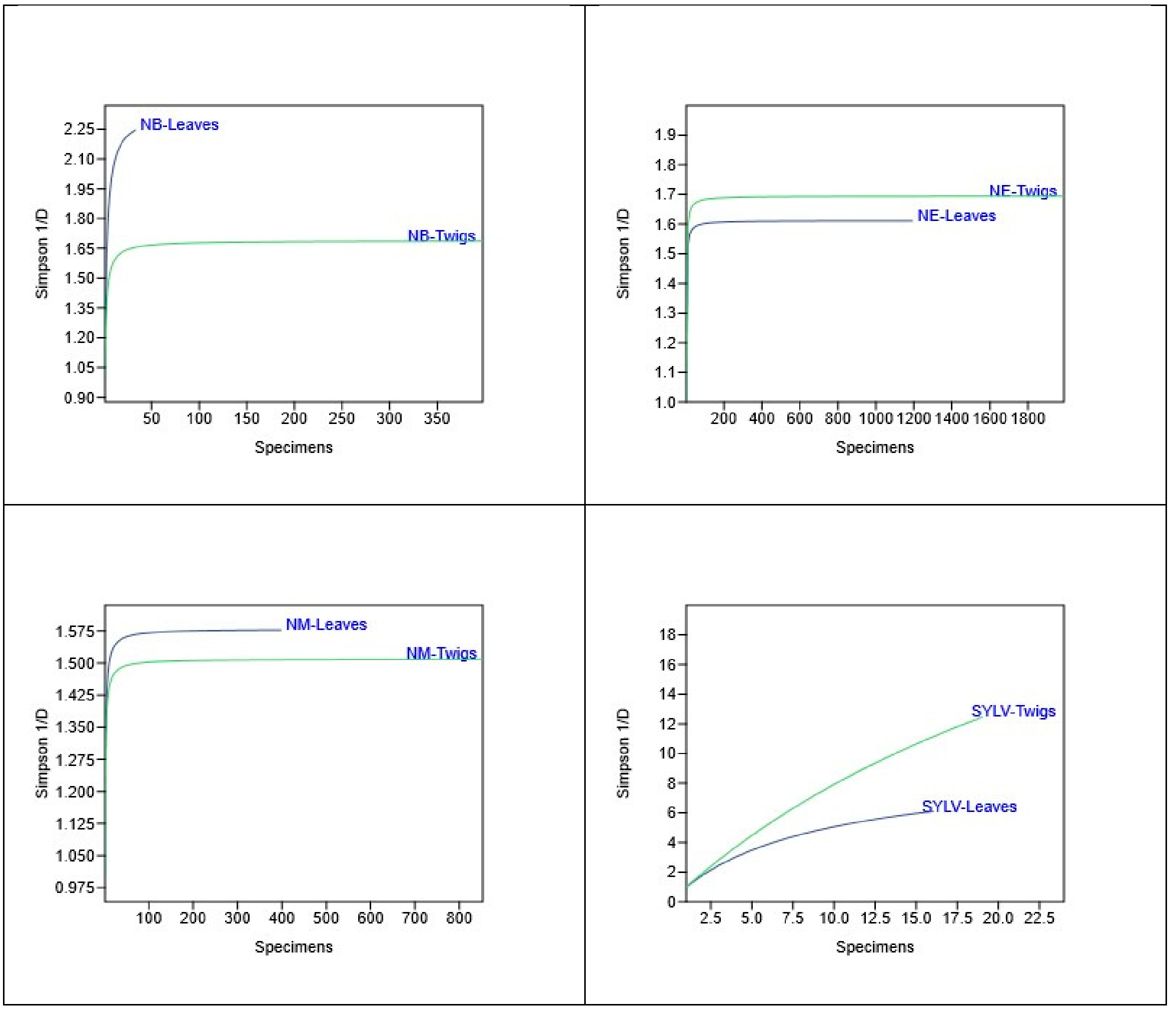

Supplement: Supplementary file 1 [file microorganisms-13-01502-s001.zip › Supplementary Figure S3_rarefaction curves.pdf]
