# Supplementary material for: Endophytic Diversity in Sicilian Olive Trees: Identifying Optimal Conditions for a Functional Microbial Collection
Source: Microorganisms. 2025 Jun 27;13(7):1502. doi: 10.3390/microorganisms13071502 (PMC12298726; doi:10.3390/microorganisms13071502)
Supplement: Supplementary file 1 [file microorganisms-13-01502-s001.zip › Supplementary Table S1_molecular ID.pdf]

Supplementary Table S1 List of identified endophytes isolated from Sicilian olive trees with the corresponding molecular identification

N.	Strain	GenBank accession number	Best BLAST match	Percent identity	Sequence length (bp)	Isolation source	Olive hosts	Number of isolates*
1	Sp_GIAL02R	PP506680	*Sphingomonas paucimobilis* strain S_WE.S7.040 (OL636271)	100%	1337	Olive twigs and leaves	GIAL03F; GIAL05F; GIAL02R; GIAL03R	4
2	Stsp_GIAL03R	PP506681	*Staphylococcus* sp. strain 9 (MN186600)	100%	1425	Olive twigs	GIAL03R; NMC03R	2
3	Bsp_NMC03R	PP506682	*Bacillus* sp. strain Zn1 (OM403112)	100%	1385	Olive twigs	NMC03R	1
4	Me_NMC03R	PP506683	*Methylorubrum extorquens* strain IMB16-167 (MG190762)	100%	1347	Olive twigs and leaves	NMC03F; NMC03R; SYLV06R	3
5	Ssp_NMC03R	PP506684	*Sphingomonas* sp. strain A3K041 (MN989146)	100%	1357	Olive twigs	NMC03R	1
6	Fsp_NMC03R	PP506685	*Frondihabitans* sp. strain A4K031 (MN989064)	100%	1379	Olive twigs	NMC03R	1
7	Bsp_NMC03F	PP506686	*Bacillus* sp. strain 1AN11 (MH712359)	99,47%	567	Olive leaves	NMC03F	1
8	Basp_NMC03R	PP506687	*Bacillus* sp. SR1 (KF896087)	100%	1385	Olive twigs	NMC03R	1
9	Stsp_NMC03R	PP506688	*Staphylococcus* sp. strain 9 (MN186600)	100%	1418	Olive twigs	GIAL03R; NMC03R	2
10	Pe_SYLV05R	PP506689	*Priestia endophytica* strain ASS-4 (OQ825037)	99,93%	1422	Olive twigs	SYLV05R	1
11	Stx_SYLV06F	PP506690	*Staphylococcus xylosus* strain PC20 (CP121162)	99,91%	1100	Olive leaves	SYLV06F	1
12	Ma_GIAL03R	PP506691	*Methylobacterium adhaesivum* strain 58g (AB698698)	98,20%	1091	Olive twigs	GIAL03R	1
13	Pp_NEC04F	PP506692	*Paenibacillus pocheonensis* strain 21918 (OR428617)	99,29%	1403	Olive leaves	NEC04F	1
14	Bm_GIAL03R	PP506693	*Priestia megaterium* strain CDC (CP069397)	100%	1424	Olive twigs	GIAL03R	1
15	Bsp_GIAL03R	PP506694	*Bacillus* sp. 210_10 (FJ938121)	100%	1421	Olive twigs	GIAL03R	1
16	Bm_GIAL02R	PP506695	*Priestia megaterium* strain CDC (CP069397)	100%	1424	Olive twigs	GIAL02R	1
17	Bsp_NMB02R	PP506696	*Bacillus* sp. strain CL2 (MK736118)	100%	1422	Olive twigs and leaves	NEB01F; NEB02F; NEB03F; NEB01R; NEB02R; NEB03R;	11

* Number of isolates with the same molecular identification derived from different olive samples.

N.	Strain	GenBank accession number	Best BLAST match	Percent identity	Sequence length (bp)	Isolation source	Olive hosts	Number of isolates*
							NMB01F; NMB02F; NMB01R; NMB02R; NMB03R	
18	Ef_GIAL02F	PP506697	*Ectobacillus funiculus* strain NAF001 (NR_028624)	99,93%	1410	Olive leaves	GIAL02F	1
19	Psav_GIAL02F	PP506698	*Pseudomonas savastanoi* strain PVFi1 (CP078139)	100%	1394	Olive leaves	GIAL02F	1
20	Bl_SYLV02R	PP506699	*Bacillus licheniformis* strain C13 (MF993022)	100%	1094	Olive twigs	SYLV02R	1
21	Sc_SYLV06R	PP506700	*Sphingomonas carotinifaciens* strain MMS22-SW14 (OP764021)	100%	1368	Olive twigs	SYLV06R	1
22	Stsp_SYLV04R	PP506701	*Staphylococcus* sp. strain 9 (MN186600)	100%	1425	Olive twigs	SYLV04R	1
23	Msp_SYLV02F	PP506702	*Methylobacterium* sp. MG-2011-68-DT (FR872484)	99.78%	1353	Olive leaves	SYLV02F	1
24	Stsp_NEB01F	PP506703	*Staphylococcus* sp. strain CAU 1474 (MG460587)	100%	1086	Olive leaves	NEB01F	1
25	Bsp_NMB01R	PP506704	*Bacillus* sp. strain 0900LM100086 (OQ874185)	100%	851	Olive twigs	NMB01R	1
26	Bsp_NEB03RIII	PP506705	*Bacillus* sp. strain HN03 (MF155192)	100%	1405	Olive twigs	NEB03R	1
27	Strsp_NMB03R	PP506706	*Streptomyces* sp. JBL-30 (AB980258)	99,78%	1379	Olive twigs	NMB03R	1
28	Strb_NMB03R	PP506707	*Streptomyces bryophytorum* strain DS3 (OP295040)	99,93%	1390	Olive twigs	NMB03R	1
29	Bsp_GIAL05R	PP506708	*Bacillus* sp. strain F-12 (MG266301)	100%	1422	Olive twigs	GIAL04F; GIAL05R	2
30	Strb_GIAL04F	PP506709	*Streptomyces bryophytorum* strain DS3 (OP295040)	100%	1397	Olive leaves	GIAL04F	1
31	Av_SYLV05R	PP506710	*Acinetobacter variabilis* strain BDT2044 (CP094246)	100%	1074	Olive twigs	SYLV05R	1
32	Bsp_NEB03RIV	PP506711	*Bacillus* sp. DY JL31 (HQ317169)	100%	1421	Olive twigs	GIAL06R; NEB03R	2
33	Bma_NMB02R	PP506712	*Bacillus marisflavi* strain BCB4-1 (MN524144)	100%	1422	Olive twigs	NMB02R	1

N.	Strain	GenBank accession number	Best BLAST match	Percent identity	Sequence length (bp)	Isolation source	Olive hosts	Number of isolates*
34	Sthae_NEC04R	PP506713	*Staphylococcus haemolyticus* strain MSA_JNM60C2 (CP065356)	100%	1394	Olive twigs	NEC04R	1
35	Stho_SYLV04R	PP506714	*Staphylococcus hominis* strain T354 (MW828139)	100%	1415	Olive twigs	SYLV04R	1
36	Stsp_NMB03F	PP506715	*Staphylococcus* sp. strain 9 (MN186600)	100%	1425	Olive leaves	NMB03F	1
37	Kp_GIAL01R	PP506716	*Kocuria palustris* strain PSRA2 (OP389235)	99,50%	1402	Olive twigs	GIAL01R	1
38	Pv_SYLV05R	PP506717	*Providencia vermicola* strain PkPS3 (MW356852)	100%	1383	Olive twigs	SYLV05R	1
39	Stsp_NMC03Ry	PV240332	*Staphylococcus* sp. strain 9 (MN186600)	100%	1417	Olive twigs	NMC03R	1
40	Bm_GIAL02Rb_B25	PV240334	*Bacillus megaterium* strain GEB3	100%	1424	Olive twigs	GIAL02R	1
41	Bsp_NMB02Ra_B23	PV240333	*Bacillus* sp. strain USAFON2	100%	1422	Olive twigs	NMB02R	1
42	Asp_GIAL04F	unknown	*Acinetobacter* sp. WB1 (KF709454)	93,28%	506	Olive leaves	GIAL04F	1
43	Pp_NMB03R	unknown	*Paenibacillus pocheonensis* strain Gsoil 1138 (NR_112565)	90,18%	713	Olive twigs	NMB03R	1
44	Bsp_NEB03R	unknown	*Bacillus* sp. strain PK3-79 (MG988225)	78,24%	1095	Olive twigs	NEB03R	1
45	Pv_NEB03R	unknown	*Providencia vermicola* strain A57 (OQ405456)	94,74%	474	Olive twigs	NEB03R	1
46	Pysp_NEB03F	PP513240	*Pyronema* sp. MAB-2010a strain CID 007 (HQ829058)	100%	538	Olive leaves	NEB03F; NMB01R; NMB02R; NMB03R	4
47	Aalt_GIAL02R	PP513241	*Alternaria alternata* isolate 1 (MW720803)	99,65%	576	Olive twigs	GIAL02R	1
48	Aalt_GIAL01R	PP513242	*Alternaria alternata* isolate 1 (MW720803)	99,65%	576	Olive twigs	GIAL01R	1
49	Cs_GIAL06F	PP513243	*Cladosporium sphaerospermum* isolate ALF6 (MW391738)	100%	494	Olive leaves	GIAL06F	1
50	Asm_GIAL03F	PP513244	*Aspergillus stella-maris* strain CBS 113639 (OL772724)	100%	517	Olive leaves	GIAL03F	1

N.	Strain	GenBank accession number	Best BLAST match	Percent identity	Sequence length (bp)	Isolation source	Olive hosts	Number of isolates*
51	Aalt_GIAL05R	PP513245	*Alternaria alternata* strain MFLUCC 16-0594 (KY026586)	100%	580	Olive twigs	GIAL05R	1
52	Pr_GIAL03R	PP513246	*Penicillium rubens* genomic DNA sequence (LT558874)	100%	585	Olive twigs	GIAL03R	1
53	Aalt_GIAL03R	PP513247	*Alternaria alternata* strain MFLUCC 16-0594 (KY026586)	100%	580	Olive twigs	GIAL03R	1
54	Qc_GIAL03RI	PP513248	*Quambalaria cyanescens* genomic DNA sequence (OW983127)	100%	651	Olive twigs	GIAL03R	1
55	Tsp_NEB02R	PP513249	*Tricharina* sp. HD-2014 isolate DO123 (KP050678)	99,24%	563	Olive twigs	NEB02R; NEB03R	2
56	Asp_NMB03R	PP513250	*Aspergillus* sp. isolate LN898694.1 (MK605981)	100%	565	Olive twigs	NMB03R	1
57	Pezsp_NEB01F	PP513251	Uncultured Pezizaceae clone 1217a (FJ788758)	96,84%	538	Olive leaves	NEB01F	1
58	Altsp_NEB01R	PP513252	*Alternaria* sp. isolate Melodinus suaveolens (MT089927)	100%	529	Olive twigs	NEB01R	1
59	Pv_NEC05R	PP513253	*Peziza varia* strain 18AJAM002 (MT520565)	100%	551	Olive twigs	NEC05R	1
60	Tstr_NMB02R	PP513254	*Tricharina striispora* voucher MV 20140222-03 (MN385979)	99,80%	539	Olive twigs	NMB02R	1
61	Stempv_NEB01R	PP513255	*Stemphylium vesicarium* isolate HST3 (MK681349)	100%	540	Olive twigs	NEB01R	1
62	Pesp_NMC03F	PP513256	*Penicillium* sp. F18 (JQ775565)	100%	558	Olive leaves	NMC03F	1
63	Tstr_NEB02R	PP513257	*Tricharina striispora* (JQ836556)	100%	511	Olive twigs	NEB02R	1
64	Phsp_NMC03R	PP513258	*Phoma* sp. isolate C 8 (KY790596)	100%	514	Olive twigs	NMC03R	1
65	Parb_NMC01R	PP513259	*Paraconiothyrium brasiliense* isolate AK (KR909140)	99,82%	568	Olive twigs	NMC01R	1
66	Parb_NMC02R	PP513260	*Paraconiothyrium brasiliense* strain HF1P01 (OP178958)	99,83%	604	Olive twigs	NMC02R	1
67	Parb_NMC03R	PP513261	*Paraconiothyrium brasiliense* (EF055358)	100%	543	Olive twigs	NMC03R	1
68	Phsp_NMC01R	PP513262	*Didymella* sp. isolate B44.2 (MN912329)	100%	507	Olive twigs	NMC01R	1

N.	Strain	GenBank accession number	Best BLAST match	Percent identity	Sequence length (bp)	Isolation source	Olive hosts	Number of isolates*
69	Altsp_NMC01R	PP513263	*Alternaria* sp. strain USGS11 (MT000591)	100%	507	Olive twigs	NMC01R	1
70	Bm_NMC02R	PP513264	*Biscogniauxia mediterranea* isolate Erm62 (MT819815)	100%	528	Olive twigs	NMC02R	1
71	Libp_NMC01F	PP513265	*Libertasomyces platani* isolate I3 (op415430)	100%	517	Olive leaves	NMC01F	1
72	Qc_NEC06R	PP513266	*Quambalaria cyanescens* culture CPC:35399 (MN162008)	100%	606	Olive twigs	NEC06R	1
73	Sosp_NEC05F	PP513267	*Sordariomycetes* sp. isolate 1T23 (MF943011)	100%	541	Olive leaves	NEC05F	1
74	Ns_NMB01R	PP513268	*Nemania serpens* strain NW-FVA2465 (MG098299)	100%	532	Olive twigs	NMB01R	1
75	Neoi_NMC02R	PP513269	*Neosetophoma italica* MFLUCC 13-0388 (NR_185358)	99,83%	595	Olive twigs	NMC02R	1
76	Df_NMC01R	PP513270	*Diaporthe foeniculina* genomic DNA sequence (LN651172)	100%	516	Olive twigs	NMC01R	1
77	Dr_SYLV02R	PP513271	*Diaporthe rudis* isolate LC5 (OL477405)	100%	591	Olive twigs	SYLV02R	1
78	Pesp_SYLV06F	PP513272	*Penicillium* sp. FPPen13 (GU270578)	100%	522	Olive leaves	SYLV06F	1
79	Eo_NMC03R	PP513273	*Elsinoe othonnae* (NR_137984)	99,82%	563	Olive twigs	NMC03R	1
80	Df_SYLV01R	PP513274	*Diaporthe foeniculina* isolate LC1 (OL477395)	100%	575	Olive twigs	SYLV01R	1
81	Prm_SYLV03F	PP513275	*Preussia minima* strain 18BAEA001 (MT645911)	100%	479	Olive leaves	SYLV03F	1
82	Bm_SYLV04R	PP513276	*Biscogniauxia mediterranea* voucher Bm10.023 (KM216762)	100%	526	Olive twigs	SYLV04R	1
83	Bm_SYLV03R	PP513277	*Biscogniauxia mediterranea* isolate Bm78 (MZ502574)	99,82%	557	Olive twigs	SYLV03R	1
84	Altsp_GIAL04R	PP513278	*Alternaria* sp. strain USGS11 (MT000591)	100%	507	Olive twigs	GIAL04R	1
85	Bm_SYLV06R	PP513279	*Biscogniauxia mediterranea* voucher Bm10.023 (KM216762)	100%	529	Olive twigs	SYLV06R	1

N.	Strain	GenBank accession number	Best BLAST match	Percent identity	Sequence length (bp)	Isolation source	Olive hosts	Number of isolates*
86	Bm_SYLV01R	PP513280	*Biscogniauxia mediterranea* voucher Bm10.023 (KM216762)	100%	523	Olive twigs	SYLV01R	1
87	Chg_SYLV05R	PP513281	*Chaetomium* sp. CBS 123940 (KC283189)	98,78%	493	Olive twigs	SYLV05R	1
88	Qc_GIAL05R	PP513282	*Quambalaria cyanescens* culture CPC:35399 (MN162008)	100%	603	Olive twigs	GIAL05R	1
89	Qc_NMC03R	PP513283	*Quambalaria cyanescens* isolate Pt6-2 (MW019647)	100%	591	Olive twigs	NMC03R	1
90	Csp_SYLV01F	PP513285	*Cladosporium* sp. isolate RS_68 (MK332482)	100%	496	Olive leaves	SYLV01F	1
91	Csp_NMC02R	PP513287	*Cladosporium* sp. isolate P1692 (KT268965)	100%	500	Olive twigs	NMC02R	1
92	Qc_NEC06F	PP513288	*Quambalaria cyanescens* culture CPC:35399 (MN162008)	100%	603	Olive leaves	NEC06F	1
93	Qc_GIAL02R	PP513289	*Quambalaria cyanescens* culture CPC:35399 (MN162008)	100%	603	Olive twigs	GIAL02R	1
94	Qc_GIAL03RIII	PP513290	*Quambalaria cyanescens* isolate Pt6-2 (MW019647)	100%	591	Olive twigs	GIAL03R	1
95	Plsp_SYLV02F	PP513291	*Pleosporineae* sp. strain 18BPLE022 (MT645935)	100%	552	Olive leaves	SYLV02F	1
96	Geosp_NMB03R	PP513293	*Geomyces* sp. 12NJ08 (JX270454)	98,65%	517	Olive twigs	NMB03R	1
97	Endp_NEC06F	PP513294	*Endoconidioma populi* isolate A39 (KX611029)	99,49%	587	Olive leaves	NEC06F	1
98	Bm_SYLV01F	PP513295	*Biscogniauxia mediterranea* isolate Bm78 (MZ502574)	100%	533	Olive leaves	SYLV01F	1
99	Penl_SYLV06R	PP513296	*Peniophora lycii* culture CBS:352.54 strain CBS 352.54 (MH857357)	99,82%	570	Olive twigs	SYLV06R	1
100	Acsc_NEB03R	PP513297	*Acremonium sclerotigenum* strain AS16 (MG583757)	100%	538	Olive twigs	NEB03R	1
101	Penc_NEB02R	PP513298	*Penicillium coffeae* strain EXK_C01 (OP592141)	100%	417	Olive twigs	NEB02R	1

N.	Strain	GenBank accession number	Best BLAST match	Percent identity	Sequence length (bp)	Isolation source	Olive hosts	Number of isolates*
102	Tritb_SYLV06F	PP513299	*Tritirachium batistae* strain URM 38 (MN496401)	99,38%	651	Olive leaves	SYLV06F	1
103	Qc_NEB02R	PP513300	*Quambalaria cyanescens* culture CPC:35399 (MN162008)	100%	603	Olive twigs	NEB02R; GIAL06R	2
104	Qc_NEC04R	PP513301	*Quambalaria cyanescens* culture CPC:35399 (MN162008)	100%	603	Olive twigs	NEC04R; NMC02R; NMC03R	3
105	Penr_SYLV06R	PP513302	*Peniophora rufomarginata* voucher CREA-DC TPR OL.178 (MW677490)	99,66%	580	Olive twigs	SYLV06R; SYLV05R	2
106	Eusp_SYLV02F	unknown	*Nothophaeomoniella ekebergiae* culture CBS:147178 (MZ064456)	94,36%	532	Olive leaves	SYLV02F	1
