# Supplementary material for: Endophytic Diversity in Sicilian Olive Trees: Identifying Optimal Conditions for a Functional Microbial Collection
Source: Microorganisms. 2025 Jun 27;13(7):1502. doi: 10.3390/microorganisms13071502 (PMC12298726; doi:10.3390/microorganisms13071502)
Supplement: Supplementary file 1 [file microorganisms-13-01502-s001.zip › Supplementary Table S3.pdf]

Supplementary Table S3 Diversity indices of endophytes associated with organic (ORG) and conventional (CONV) farming system of different cultivated olive hosts (NB, Nocellara del Belice; NE, Nocellara etnea; NM, Nocellara messinese).

Diversity indexes	NB-Org	NB-Conv	NE-Org	NE-Conv	NM-Org	NM-Conv
Taxa (genera)	12	7	10	6	10	15
Individuals	98	333	3191	15	1020	230
Dominance	0.211	0.831	0.585	0.429	0.800	0.199
Simpson (1-D)	0.790	0.169	0.415	0.571	0.200	0.801
Shannon	1.911	0.406	0.866	1.340	0.444	1.987
Equitability	0.770	0.209	0.376	0.748	0.193	0.734
Chao-1	12.99	12.98	16	15.33	13	19.98
