# Supplementary material for: Endophytic Diversity in Sicilian Olive Trees: Identifying Optimal Conditions for a Functional Microbial Collection
Source: Microorganisms. 2025 Jun 27;13(7):1502. doi: 10.3390/microorganisms13071502 (PMC12298726; doi:10.3390/microorganisms13071502)
Supplement: Supplementary file 1 [file microorganisms-13-01502-s001.zip › Supplementary Table S4.pdf]

Supplementary Table S4. PGP properties showed by the endophytes isolated from Sicilian olive tree. Selected strains to dual-culture test appear in bold.

Isolate	Identification	IAA production	Siderophore production	Phosphate solubilisation	Nitrogen fixation	Biofilm	ACC deaminase activity
**Sp_GIAL02R**	***Sphingomonas paucimobilis***	**1.29**	**0.2**	**0.1**	**-**	**++**	**-**
Stsp_GIAL03R	*Staphylococcus* sp.	0.17	1.0	-	-	+	-
Asp_GIAL04F	unknown	-	0.5	-	+	++	-
**Bsp_NMC03R**	***Bacillus* sp.**	**1.38**	**-**	**-**	**+**	**++**	**-**
Me_NMC03R	*Methylorubrum extorquens*	-	-	-	+	+	-
Ssp_NMC03R	*Sphingomonas* sp.	-	0.4	-	-	n.a	n.a
Fsp_NMC03R	*Frondihabitans* sp.	-	-	-	-	n.a	-
Bsp_NMC03F	*Bacillus* sp.	-	1.4	-	-	n.a	n.a
Basp_NMC03R	*Bacillus* sp.	-	2.5	-	+	++	-
Stsp_NMC03Ry	*Staphylococcus* sp.	-	-	-	+	+	-
Stsp_NMC03R	*Staphylococcus* sp.	-	1.6	0.1	+	-	-
**Pe_SYLV05R**	***Priestia endophytica***	**6.18**	**1.0**	**-**	**+**	**+**	**-**
Pp_NEC04F	*Paenibacillus pocheonensis*	-	-	0,1	+	+	-
Pv_NEB03R	unknown	-	0.2	-	-	+	-
Bm_GIAL03R	*Bacillus megaterium*	-	0.3	-	-	-	-
Bsp_GIAL03R	*Bacillus* sp.	-	0.1	-	+	+	-
Bm_GIAL02R	*Bacillus megaterium*	0.26	2.5	0.2	+	+	-
Bsp_NMB02R	*Bacillus* sp.	0.21	1.0	-	+	+	-
Bsp_NMB02Ra	*Bacillus* sp.	-	0,1	-	+	++	-
Ef_GIAL02F	*Ectobacillus funiculus*	0.17	-	0.1	+	+	-
Bm_GIAL02Rb	*Bacillus megaterium*	0.95	2.2	-	+	+	-
Psav_GIAL02F	*Pseudomonas savastanoi*	2.71	-	-	weakly+	-	-
**Bl_SYLV02R**	***Bacillus licheniformis***	**0.33**	**1.7**	**-**	**+**	**++**	**-**
Bsp_NEB03R	unknown	-	1.3	-	+	-	-
Sc_SYLV06R	*Sphingomonas carotinifaciens*	-	-	-	+	-	-
Stsp_SYLV04R	*Staphylococcus* sp.	2.31	0.2	-	-	+	-
Msp_SYLV02F	*Methylobacterium* sp.	-	0.5	0.2	-	+	-
Stsp_NEB01F	*Staphylococcus* sp.	-	1.2	0.1	+	+	-
Pp_NMB03R	unknown	-	2.0	-	+	+	-
Bsp_NMB01R	*Bacillus* sp.	-	1.1	-	+	+	-
Bsp_NEB03RIII	*Bacillus* sp.	-	1.8	-	+	+	-
Bsp_GIAL05R	*Bacillus* sp.	-	3.0	-	+	+	-
Av_SYLV05R	*Acinetobacter variabilis*	0.12	0.4	-	weakly+	+	-
Bsp_NEB03RIV	*Bacillus* sp.	-	1.0	-	+	+	-
**Bma_NMB02R**	***Bacillus marisflavi***	**3.64**	**1.8**	**-**	weakly+	**-**	**-**
Sthae_NEC04R	*Staphylococcus haemolyticus*	-	0.3	-	-	+	-
Stho_SYLV04R	*Staphylococcus hominis*	2.41	0.2	-	-	+	-
Stsp_NMB03F	*Staphylococcus* sp.	0.24	0.2	-	-	+	-
**Pv_SYLV05R**	***Providencia vermicola***	**9.74**	**1.0**	**0.2**	**-**	**++**	**-**
Kp_GIAL01R	*Kocuria palustris*	-	0.2	-	-	++	-

+, presence of the activity; weakly+, moderate activity*; -, absence of the activity. Values of phosphate solubilisation and siderophores production express the diameter of the halo in cm. Values of IAA production are expressed in mg·L^{-1}. Values of ACC deaminase activity are expressed in μmoles α-ketobutyrate·mg protein^{-1}·h^{-1}

* It indicates an outcome that is neither completely negative nor fully positive, but falls somewhere in between, with encouraging trends.
