# Supplementary material for: Endophytic Diversity in Sicilian Olive Trees: Identifying Optimal Conditions for a Functional Microbial Collection
Source: Microorganisms. 2025 Jun 27;13(7):1502. doi: 10.3390/microorganisms13071502 (PMC12298726; doi:10.3390/microorganisms13071502)
Supplement: Supplementary file 1 [file microorganisms-13-01502-s001.zip › Supplementary Table S5 (Enzimatic activity results).pdf]

Supplementary Table S5. Enzymatic activities showed by the endophytes isolated from Sicilian olive tree. Selected strains to dual-culture test appear in bold.

Isolate	Identification	DNAse	Amylase	Lipase	Cellulase	Pectinase	Protease	Chitinase
**Sp_GIAL02R**	***Sphingomonas paucimobilis***	**+**	**-**	**+**	**weakly+**	**-**	**-**	**-**
Stsp_GIAL03R	*Staphylococcus* sp.	-	-	-	-	-	-	-
Asp_GIAL04F	unknown	+	-	+	-	-	-	-
**Bsp_NMC03R**	***Bacillus* sp.**	**++**	**weakly+**	**+**	**weakly+**	**-**	**++**	**+**
Me_NMC03R	*Methylorubrum extorquens*	-	+++	+	-	-	-	-
Ssp_NMC03R	*Spingomonas* sp.	++	weakly+	+	-	-	-	-
Fsp_NMC03R	*Frondihabitans* sp.	weakly+	+	+	-	-	-	-
Bsp_NMC03F	*Bacillus* sp.	weakly+	+++	+++	-	++	++	-
Basp_NMC03R	*Bacillus* sp.	++	-	-	-	-	+	-
Stsp_NMC03Ry	*Staphylococcus* sp.	-	weakly+	++	-	-	-	-
Stsp_NMC03R	*Staphylococcus* sp.	-	+++	-	-	-	-	-
**Pe_SYLV05R**	***Priestia endophytica***	**-**	**weakly+**	**-**	**-**	**+**	**-**	**-**
Pp_NEC04F	*Paenibacillus pocheonensis*	weakly+	++	-	-	+	-	-
Pv_NEB03R	unknown	weakly+	-	weakly+	-	-	-	-
Bm_GIAL03R	*Bacillus megaterium*	+	-	-	-	-	-	-
Bsp_GIAL03R	*Bacillus* sp.	++	+	-	-	-	+	-
Bm_GIAL02R	*Bacillus megaterium*	-	++	-	-	-	-	-
Bsp_NMB02R	*Bacillus* sp.	-	++	weakly+	+	-	+	-
Bsp_NMB02Ra	*Bacillus* sp.	-	+	weakly+	-	+	+	-
Ef_GIAL02F	*Ectobacillus funiculus*	-	++	+	-	-	-	-
Bm_GIAL02Rb	*Bacillus megaterium*	-	++	-	-	-	++	-
Psav_GIAL02F	*Pseudomonas savastanoi*	+	+	-	-	-	+	-
**Bl_SYLV02R**	***Bacillus licheniformis***	**++**	**++**	**-**	**+**	**+**	**+**	**+**
Bsp_NEB03R	unknown	weakly+	++	++	-	-	-	-
Sc_SYLV06R	*Sphingomonas carotinifaciens*	-	-	+	-	-	-	-
Stsp_SYLV04R	*Staphylococcus* sp.	weakly+	-	+	-	-	-	-
Msp_SYLV02F	*Methylobacterium* sp.	-	-	-	-	-	+	-
Stsp_NEB01F	*Staphylococcus* sp.	-	-	++	-	-	-	-
Pp_NMB03R	unknown	-	++	-	-	-	-	-
Bsp_NMB01R	*Bacillus* sp.	-	-	weakly+	weakly+	weakly+	+	-
Bsp_NEB03RIII	*Bacillus* sp.	+	-	+	-	-	++	-
Bsp_GIAL05R	*Bacillus* sp.	++	+	-	-	-	+	-
Av_SYLV05R	*Acinetobacter variabilis*	-	-	++	-	-	-	-
Bsp_NEB03RIV	*Bacillus* sp.	-	++	+	weakly+	-	+	-
**Bma_NMB02R**	***Bacillus marisflavi***	**++**	**+**	**-**	**-**	**weakly+**	**+**	**-**
Sthae_NEC04R	*Staphylococcus haemolyticus*	+	-	-	-	-	-	-
Stho_SYLV04R	*Staphylococcus hominis*	weakly+	-	-	-	-	-	-
Stsp_NMB03F	*Staphylococcus* sp.	weakly+	-	+	-	-	-	-
**Pv_SYLV05R**	***Providencia vermicola***	**-**	**-**	**-**	**-**	**-**	**-**	**++**
Kp_GIAL01R	*Kocuria palustris*	-	+	-	-	-	-	-

+, presence of the activity; weakly+, moderate activity*; -, absence of the activity.

* It indicates an outcome that is neither completely negative nor fully positive, but falls somewhere in between, with encouraging trends.
