# Supplementary material for: Endophytic Diversity in Sicilian Olive Trees: Identifying Optimal Conditions for a Functional Microbial Collection
Source: Microorganisms. 2025 Jun 27;13(7):1502. doi: 10.3390/microorganisms13071502 (PMC12298726; doi:10.3390/microorganisms13071502)
Supplement: Supplementary file 1 [file microorganisms-13-01502-s001.zip › Supplementary Table S6 (antagonistic activity).pdf]

Supplementary Table S6 Antagonistic ability of endophyte strains and positive control (lines) against fungal pathogens (columns). Antifungal activities are reported as percentages of mycelial growth inhibition.

**Bacterial endophytes *vs* fungal pathogens**	*Neofusicoccum parvum* P4	*Neofusicoccum vitifusiforme* P3	*Verticillium dahliae* CZ	*Verticillium dahliae* CS
Bacillus sp. Bsp_NMC03R	-	-	-	-
Bacillus licheniformis Bl_SYLV02R	-	40.3%	-	-
Providencia vermicola Pv_SYLV05R	-	-	-	-
Sphingomonas paucimobilis Sp_GIAL02R	-	-	-	-
Priestia endophytica Pe_SYLV05R	-	-	-	-
Bacillus marisflavi Bma_NMB02R	-	-	-	-
Trichoderma harzianum S3	54.05%	60%	37.9%	25%
