# Supplementary material for: Endophytic Diversity in Sicilian Olive Trees: Identifying Optimal Conditions for a Functional Microbial Collection
Source: Microorganisms. 2025 Jun 27;13(7):1502. doi: 10.3390/microorganisms13071502 (PMC12298726; doi:10.3390/microorganisms13071502)
Supplement: Supplementary file 1 [file microorganisms-13-01502-s001.zip › Supplementary Table S7 (FUNGuild).pdf]

Supplementary Table S7. Functional diversity of fungal endophytes isolated from Sicilian olive tree, according to FUNGuild database.

Number	Isolate	GenBank accession number	Identification	Putative trophism on FUNGUILD	Guild
1	Pysp_NEB03F	PP513240	*Pyronema* sp.	Saprotroph-Symbiotroph	Endophyte-Plant Saprotroph-Undefined Saprotroph
2	Aalt_GIAL02R	PP513241	*Alternaria alternata*	Pathotroph-Saprotroph-Symbiotroph	Animal Pathogen-Endophyte-Plant Pathogen-Wood Saprotroph
3	Aalt_GIAL01R	PP513242	*Alternaria alternata*	Pathotroph-Saprotroph-Symbiotroph	Animal Pathogen-Endophyte-Plant Pathogen-Wood Saprotroph
4	Cs_GIAL06F	PP513243	*Cladosporium sphaerospermum*	Pathotroph-Saprotroph-Symbiotroph	Animal Pathogen-Endophyte-Lichen Parasite-Plant Pathogen-Plant Saprotroph-Wood Saprotroph
5	Asm_GIAL03F	PP513244	*Aspergillus stella-maris*	Pathotroph-Saprotroph-Symbiotroph	Animal Pathogen-Endophyte-Plant Saprotroph-Undefined Saprotroph-Wood Saprotroph
6	Aalt_GIAL05R	PP513245	*Alternaria alternata*	Pathotroph-Saprotroph-Symbiotroph	Animal Pathogen-Endophyte-Plant Pathogen-Wood Saprotroph
7	Pr_GIAL03R	PP513246	*Penicillium rubens*	Pathotroph-Saprotroph-Symbiotroph	Animal Parasite-Dung Saprotroph-Endophyte-Fungal Parasite-Plant Pathogen-Undefined Saprotroph-Wood Saprotroph
8	Aalt_GIAL03R	PP513247	*Alternaria alternata*	Pathotroph-Saprotroph-Symbiotroph	Animal Pathogen-Endophyte-Plant Pathogen-Wood Saprotroph
9	Qc_GIAL03RI	PP513248	*Quambalaria cyanescens*	Pathotroph	Plant Pathogen
10	Tsp_NEB02R	PP513249	*Tricharina* sp.	Saprotroph-Symbiotroph	Endophyte-Undefined Saprotroph
11	Asp_NMB03R	PP513250	*Aspergillus* sp.	Pathotroph-Saprotroph-Symbiotroph	Animal Pathogen-Endophyte-Plant Saprotroph-Undefined Saprotroph-Wood Saprotroph
12	Pezsp_NEB01F	PP513251	Uncultured Pezizaceae	Saprotroph-Symbiotroph	Dung Saprotroph-Ectomycorrhizal-Litter Saprotroph-Undefined Saprotroph

Number	Isolate	GenBank accession number	Identification	Putative trophism on FUNGUILD	Guild
13	Altsp_NEB01R	PP513252	*Alternaria* sp.	Pathotroph-Saprotroph-Symbiotroph	Animal Pathogen-Endophyte-Plant Pathogen-Plant Saprotroph-Wood Saprotroph
14	Pv_NEC05R	PP513253	*Peziza varia*	Saprotroph-Symbiotroph	Dung Saprotroph-Ectomycorrhizal-Endophyte-Plant Saprotroph-Undefined Saprotroph
15	Tstr_NMB02R	PP513254	*Tricharina striispora*	Saprotroph-Symbiotroph	Endophyte-Undefined Saprotroph
16	Stempv_NEB01R	PP513255	*Stemphylium vesicarium*	Pathotroph-Saprotroph	Plant Pathogen-Plant Saprotroph-Wood Saprotroph
17	Pesp_NMC03F	PP513256	*Penicillium* sp.	Pathotroph-Saprotroph-Symbiotroph	Animal Parasite-Dung Saprotroph-Endophyte-Fungal Parasite-Plant Pathogen-Undefined Saprotroph-Wood Saprotroph
18	Tstr_NEB02R	PP513257	*Tricharina striispora*	Saprotroph-Symbiotroph	Endophyte-Undefined Saprotroph
19	Phsp_NMC03R	PP513258	*Phoma* sp.	Pathotroph-Saprotroph-Symbiotroph	Dung Saprotroph-Endophyte-Lichen Parasite-Plant Pathogen-Plant Saprotroph-Undefined Saprotroph-Wood Saprotroph
20	Parb_NMC01R	PP513259	*Paraconiothyrium brasiliense*	Saprotroph	Undefined Saprotroph-Wood Saprotroph
21	Parb_NMC02R	PP513260	*Paraconiothyrium brasiliense*	Saprotroph	Undefined Saprotroph-Wood Saprotroph
22	Parb_NMC03R	PP513261	*Paraconiothyrium brasiliense*	Saprotroph	Undefined Saprotroph-Wood Saprotroph
23	Phsp_NMC01R	PP513262	*Didymella* sp.	Pathotroph-Saprotroph	Animal Pathogen-Plant Pathogen-Plant Saprotroph-Undefined Saprotroph
24	Altsp_NMC01R	PP513263	*Alternaria* sp.	Pathotroph-Saprotroph-Symbiotroph	Animal Pathogen-Endophyte-Plant Pathogen-Plant Saprotroph-Wood Saprotroph
25	Bm_NMC02R	PP513264	*Biscogniauxia mediterranea*	Pathotroph-Saprotroph-Symbiotroph	Endophyte-Plant Pathogen-Undefined Saprotroph-Wood Saprotroph
26	Libp_NMC01F	PP513265	*Libertasomyces platani*	Saprotroph	Plant Saprotroph

Number	Isolate	GenBank accession number	Identification	Putative trophism on FUNGUILD	Guild
27	Qc_NEC06R	PP513266	*Quambalaria cyanescens*	Pathotroph	Plant Pathogen
28	Sosp_NEC05F	PP513267	*Sordariomycetes* sp.	No data available	No data available
29	Ns_NMB01R	PP513268	*Nemania serpens*	Saprotroph	Undefined Saprotroph-Wood Saprotroph
30	Neoi_NMC02R	PP513269	*Neosetophoma italica*	Saprotroph-Symbiotroph	Endophyte-Plant Saprotroph-Undefined Saprotroph
31	Df_NMC01R	PP513270	*Diaporthe foeniculina*	Pathotroph-Saprotroph-Symbiotroph	Endophyte-Plant Pathogen-Plant Saprotroph
32	Dr_SYLV02R	PP513271	*Diaporthe rudis*	Pathotroph-Saprotroph-Symbiotroph	Endophyte-Plant Pathogen-Plant Saprotroph
33	Pesp_SYLV06F	PP513272	*Penicillium* sp.	Pathotroph-Saprotroph-Symbiotroph	Animal Parasite-Dung Saprotroph-Endophyte-Fungal Parasite-Plant Pathogen-Undefined Saprotroph-Wood Saprotroph
34	Eo_NMC03R	PP513273	*Elsinoe othonnae*	Pathotroph-Saprotroph	Plant Pathogen-Plant Saprotroph
35	Df_SYLV01R	PP513274	*Diaporthe foeniculina*	Pathotroph-Saprotroph-Symbiotroph	Endophyte-Plant Pathogen-Plant Saprotroph
36	Prm_SYLV03F	PP513275	*Preussia minima*	Saprotroph	Dung Saprotroph-Undefined Saprotroph
37	Bm_SYLV04R	PP513276	*Biscogniauxia mediterranea*	Pathotroph-Saprotroph-Symbiotroph	Endophyte-Plant Pathogen-Undefined Saprotroph-Wood Saprotroph
38	Bm_SYLV03R	PP513277	*Biscogniauxia mediterranea*	Pathotroph-Saprotroph-Symbiotroph	Endophyte-Plant Pathogen-Undefined Saprotroph-Wood Saprotroph
39	Altsp_GIAL04R	PP513278	*Alternaria* sp.	Pathotroph-Saprotroph-Symbiotroph	Animal Pathogen-Endophyte-Plant Pathogen-Plant Saprotroph-Wood Saprotroph
40	Bm_SYLV06R	PP513279	*Biscogniauxia mediterranea*	Pathotroph-Saprotroph-Symbiotroph	Endophyte-Plant Pathogen-Undefined Saprotroph-Wood Saprotroph
41	Bm_SYLV01R	PP513280	*Biscogniauxia mediterranea*	Pathotroph-Saprotroph-Symbiotroph	Endophyte-Plant Pathogen-Undefined Saprotroph-Wood Saprotroph
42	Chg_SYLV05R	PP513281	*Chaetomium* sp.	Pathotroph-Saprotroph-Symbiotroph	Animal Pathogen-Dung Saprotroph-Endophyte-Epiphyte-Plant Pathogen-Plant Saprotroph-Wood Saprotroph

Number	Isolate	GenBank accession number	Identification	Putative trophism on FUNGUILD	Guild
43	Qc_GIAL05R	PP513282	*Quambalaria cyanescens*	Pathotroph	Plant Pathogen
44	Qc_NMC03R	PP513283	*Quambalaria cyanescens*	Pathotroph	Plant Pathogen
45	Csp_SYLV01F	PP513285	*Cladosporium* sp.	Pathotroph-Saprotroph-Symbiotroph	Animal Pathogen-Endophyte-Lichen Parasite-Plant Pathogen-Plant Saprotroph-Wood Saprotroph
46	Csp_NMC02R	PP513287	*Cladosporium* sp.	Pathotroph-Saprotroph-Symbiotroph	Animal Pathogen-Endophyte-Lichen Parasite-Plant Pathogen-Plant Saprotroph-Wood Saprotroph
47	Qc_NEC06F	PP513288	*Quambalaria cyanescens*	Pathotroph	Plant Pathogen
48	Qc_GIAL02R	PP513289	*Quambalaria cyanescens*	Pathotroph	Plant Pathogen
49	Qc_GIAL03RIII	PP513290	*Quambalaria cyanescens*	Pathotroph	Plant Pathogen
50	Plsp_SYLV02F	PP513291	*Pleosporineae* sp.	No data available	No data available
51	Geosp_NMB03R	PP513293	*Geomyces* sp.	Saprotroph	Undefined Saprotroph
52	Endp_NEC06F	PP513294	*Endoconidioma populi*	Saprotroph	Undefined Saprotroph-Wood Saprotroph
53	Bm_SYLV01F	PP513295	*Biscogniauxia mediterranea*	Pathotroph-Saprotroph-Symbiotroph	Endophyte-Plant Pathogen-Undefined Saprotroph-Wood Saprotroph
54	Penl_SYLV06R	PP513296	*Peniophora lycii*	Pathotroph-Saprotroph	Plant Pathogen-Wood Saprotroph
55	Acsc_NEB03R	PP513297	*Acremonium sclerotigenum*	Pathotroph-Saprotroph-Symbiotroph	Animal Pathogen-Endophyte-Fungal Parasite-Plant Pathogen-Undefined Saprotroph-Wood Saprotroph
56	Penc_NEB02R	PP513298	*Penicillium coffeae*	Pathotroph-Saprotroph-Symbiotroph	Animal Parasite-Dung Saprotroph-Endophyte-Fungal Parasite-Plant Pathogen-Undefined Saprotroph-Wood Saprotroph
57	Tritb_SYLV06F	PP513299	*Tritirachium batistae*	Pathotroph-Saprotroph	Animal Parasite-Undefined Saprotroph
58	Qc_NEB02R	PP513300	*Quambalaria cyanescens*	Pathotroph	Plant Pathogen

Number	Isolate	GenBank accession number	Identification	Putative trophism on FUNGUILD	Guild
59	Qc_NEC04R	PP513301	*Quambalaria cyanescens*	Pathotroph	Plant Pathogen
60	Penr_SYLV06R	PP513302	*Peniophora rufomarginata*	Pathotroph-Saprotroph	Plant Pathogen-Wood Saprotroph
61	Eusp_SYLV02F	unknown	*Nothophaeomoniella ekebergiae*	No data available	No data available
