# Supplementary material for: Endophytic Diversity in Sicilian Olive Trees: Identifying Optimal Conditions for a Functional Microbial Collection
Source: Microorganisms. 2025 Jun 27;13(7):1502. doi: 10.3390/microorganisms13071502 (PMC12298726; doi:10.3390/microorganisms13071502)
Supplement: Supplementary file 1 [file microorganisms-13-01502-s001.zip › Supplementary Tables S2.pdf]

Supplementary Tables S2 Diversity indices of endophytes associated with leaves (L) and twigs (T) of different olive hosts (NB, Nocellara del Belice; NE, Nocellara etnea; NM, Nocellara messinese; SYLV, wild olive accessions) during the four phenological phases studied (WD, winter rest; FL, flowering; FS, fruit set; MAT, fruit maturation).

	WD		WD		WD		WD	
**Diversity indexes**	**NB-L**	**NB-T**	**NE-L**	**NE-T**	**NM-L**	**NM-T**	**SYLV-L**	**SYLV-T**
Taxa (genera)	3	6	4	6	3	16	3	5
Individuals	28	81	303	9	3	208	3	7
Dominance	0.595	0.291	0.980	0.111	0	0.223	0	0.095
Simpson (1-D)	0.405	0.709	0.019	0.889	1	0.777	1	0.905
Shannon	0.701	1.427	0.071	1.955	1.432	1.945	1.432	1.836
Equitability	0.638	0.797	0.051	1.091	1.303	0.701	1.303	1.140
Chao-1	3	6	6.99	8.67	5	23.46	5	5.857

	FL		FL		FL		FL	
**Diversity indexes**	**NB-L**	**NB-T**	**NE-L**	**NE-T**	**NM-L**	**NM-T**	**SYLV-L**	**SYLV-T**
Taxa (genera)	2	4	1	1	1	1	3	6
Individuals	2	6	900	900	302	605	9	6
Dominance	0	0.133	1	1	1	1	0.333	0
Simpson (1-D)	1	0.867	0	0	0	0	0.667	1
Shannon	0.943	1.580	0	0	0	0	1.076	2.208
Equitability	1.361	1.139	-	-	-	-	0.9795	1.233
Chao-1	2.5	4.3	1	1	1	1	3	18.5

	FS		FS		FS		FS	
**Diversity indexes**	**NB-L**	**NB-T**	**NE-L**	**NE-T**	**NM-L**	**NM-T**	**SYLV-L**	**SYLV-T**
Taxa (genera)	2	2	1	2	1	5	1	n.a
Individuals	3	5	1	475	2	32	2	n.a
Dominance	0.333	0.6	-	0.534	1	0.571	1	n.a
Simpson (1-D)	0.667	0.4	-	0.466	0	0.429	0	n.a
Shannon	0.803	0.600	0	0.659	0	0.928	0	n.a
Equitability	1.159	0.866	-	0.951	-	0.577	-	n.a
Chao-1	2.3	2	1	2	1	5.48	1	n.a

	MAT		MAT		MAT		MAT	
**Diversity indexes**	**NB-L**	**NB-T**	**NE-L**	**NE-T**	**NM-L**	**NM-T**	**SYLV-L**	**SYLV-T**
Taxa (genera)	n.a	3	n.a	5	1	2	2	5
Individuals	n.a	306	n.a	618	90	8	2	6
Dominance	n.a	0.961	n.a	0.361	1	0.750	0	0.067
Simpson (1-D)	n.a	0.039	n.a	0.639	0	0.250	1	0.933
Shannon	n.a	0.109	n.a	1.141	0	0.439	0.943	1.894
Equitability	n.a	0.099	n.a	0.709	-	0.634	1.361	1.177
Chao-1	n.a	3	n.a	5	1	2	2.5	7.5
