# Supplementary figures and images for: Endophytic Diversity in Sicilian Olive Trees: Identifying Optimal Conditions for a Functional Microbial Collection
Source: Microorganisms. 2025 Jun 27;13(7):1502. doi: 10.3390/microorganisms13071502 (PMC12298726; doi:10.3390/microorganisms13071502)

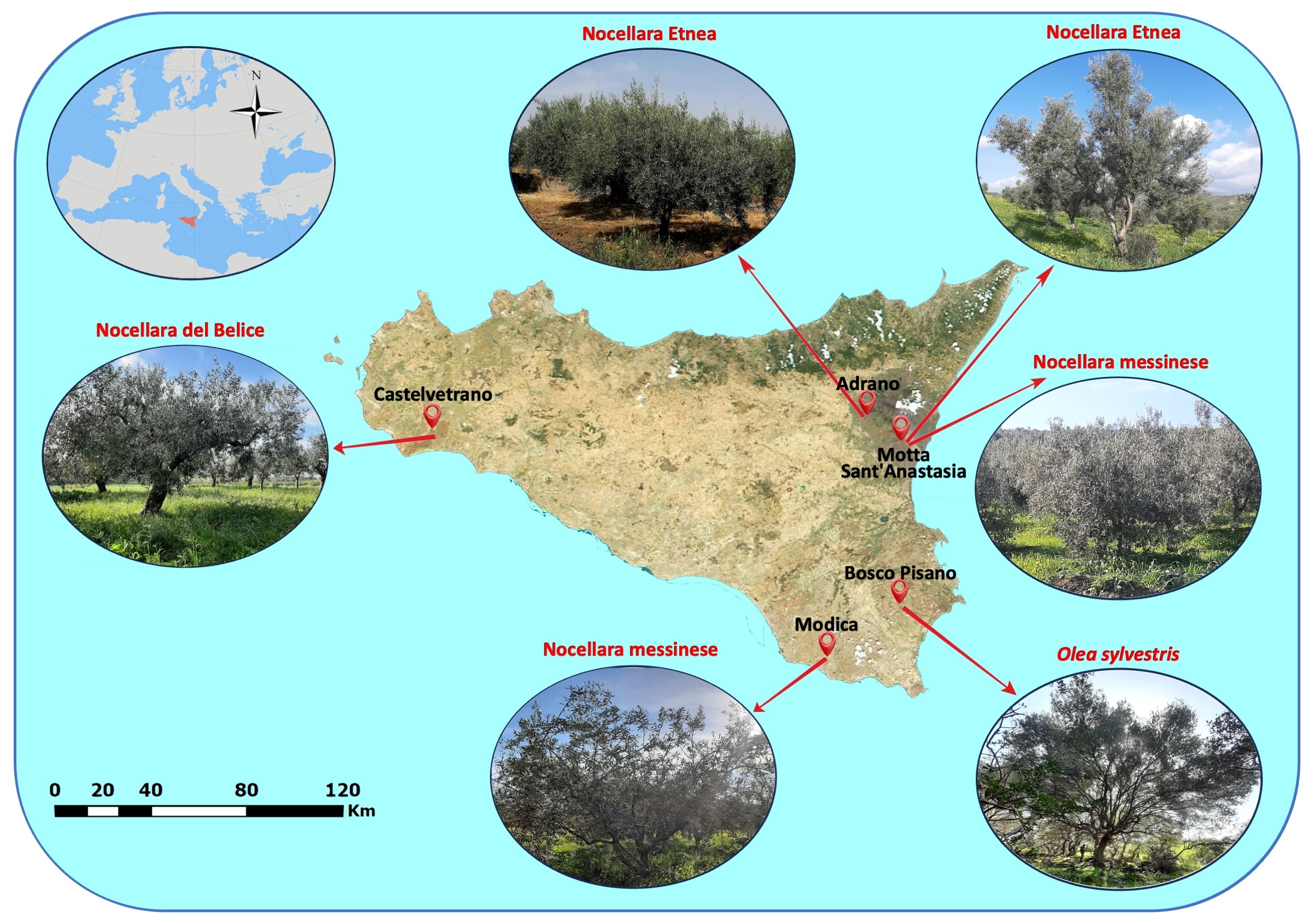

Supplement: Supplementary file 1 [file microorganisms-13-01502-s001.zip › Supplementary Figure S1 (immagine campionamento).jpg]
